# Factors to Consider for Synthesis in 1536-Well Plates—An Amide Coupling Case Study for PROTAC Synthesis

**DOI:** 10.1021/acs.joc.4c02456

**Published:** 2025-02-03

**Authors:** Rebecca Stevens, Harry E. P. Palmer, Afjal H. Miah, Glenn A. Burley

**Affiliations:** † Modality Platform Technologies, GSK, Stevenage SG1 2NY, U.K.; ‡ Department of Pure and Applied Chemistry, University of Strathclyde, Glasgow G1 1BX, U.K.

## Abstract

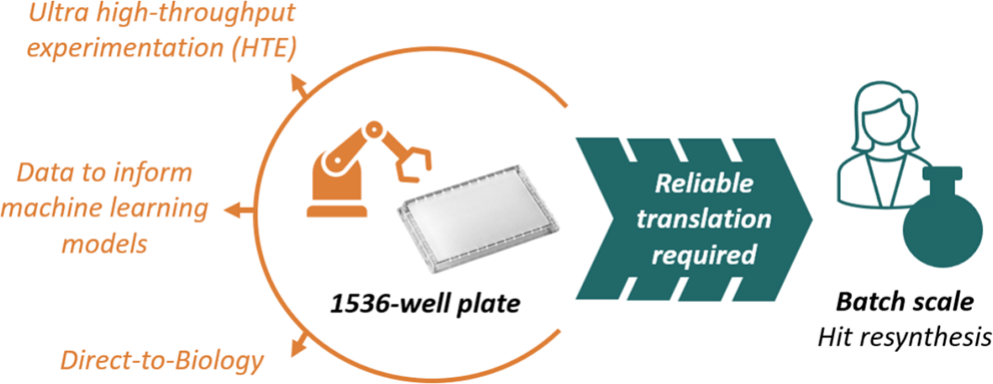

Ultra high-throughput chemistry carried out in 1536-well plates is increasingly utilized for reaction optimization protocols and direct-to-biology (D2B) platforms, where nanomolar quantities of the final product are directly assessed for biochemical or cellular activity without purification. As their popularity increases, it is crucial that the synthesis of these molecules is reliable and reproducible. Research in our laboratories has identified several nuances of amide couplings when performed on the nanoscale that result in poor translation from 1536-well plates to batch-scale reactions. This case study presents a nanoscale amide coupling reaction to synthesize 700 PROTAC molecules, which identified a range of factors crucial to reaction success on the nanoscale, despite having no influence on reaction conversion in batch. This work presents a guide for high-throughput chemists to consider when working in 1536-well plates and their importance in drawing conclusions from nanoscale synthesis.

## Introduction

High-throughput chemistry is an enabling tool in the pharmaceutical and biotechnology industries.^1^ With a range of protocols first developed for biology procedures, high-throughput screening (HTS) has been widely used for several decades to identify hit molecules for a target of interest.^2−4^ High-throughput approaches are now being employed in later stages of the drug discovery process, for example, reaction optimization in synthetic or medicinal chemistry groups is routinely carried out using high-throughput experimentation (HTE) in 96-, 384- or 1536-well plates.^5−7^ Example applications include transition metal catalysis; hydrogenations and challenging cross couplings;^8−10^ reactions on highly functionalized drug leads;^11^ and biocatalytic transformations.^12−14^ These protocols are frequently aided by automation tools such as advanced liquid handling equipment.^15^ Process chemistry departments have also reported the use of HTE for the development of workup conditions,^16^ chiral salt resolution, and scavenger screening.^12^

Alongside reaction optimization, high-throughput plate-based synthesis has found direct application to the drug discovery process in the use of direct-to-biology (D2B) platforms, where compounds synthesized in microtiter plates are directly evaluated for biological activity in biochemical or cellular assays without purification. D2B approaches have been employed in the discovery of molecular glues,^17,18^ PROTACs,^19−23^ kinase inhibitors,^24^ and reactive fragments,^25^ and offer the opportunity to accelerate discovery, avoiding the bottleneck of compound purification, while reducing the mass of precious advanced intermediates required to make analogues.

The use of HTE has led to a marked increase in the availability of reaction data, and in recent years, there has been a focus on using data from high-throughput workflows as training sets for machine learning or artificial intelligence (AI).^26−28^ The use of automation may reduce the number of false positive or negative data points that occur due to human error, but if HTE data are employed to inform these models, a clear understanding of translatability between plate-based workflows and batch scale is crucial for chemists using these tools.

While efforts to improve reproducibility have been made by enabling the use of homogeneous reaction mixtures,^29−31^ and there are examples where excellent translation from 1536-well plates to batch scale has been exemplified,^7,11^ negative data is not typically reported in the scientific literature. In this article, we demonstrate the challenges associated with the reproducibility of reactions performed on the nanoscale. We show that the reactivity of amide coupling reactions deviates when performed in a plate-based format relative to batch-based processes. We propose a set of rules to assist the chemist when performing reaction optimization or D2B in a 1536-well plate format.

## Results and Discussion

This study began when we observed a deviation in the outcome of a series of amide couplings to synthesize proteolysis targeting chimeras (PROTACs) in 1536-well plates.

PROTACs are heterobifunctional molecules composed of two ligands, one for a protein-of-interest (POI) and one for an E3 ligase, connected via a linker. By binding of the ligands to their respective protein partners, the PROTAC induces a ternary complex, leading to proximity-induced ubiquitination of the POI, and subsequent targeted protein degradation by the ubiquitin proteasome system (UPS).

Our previously reported conditions for plate-based PROTAC synthesis using EDC, OxymaPure, and NMM in 5 μL of DMSO were employed as these were amenable to D2B, where evaluation of the reaction mixtures could be carried out in a cellular HiBiT assay without any purification.^21^

**Scheme 1:**
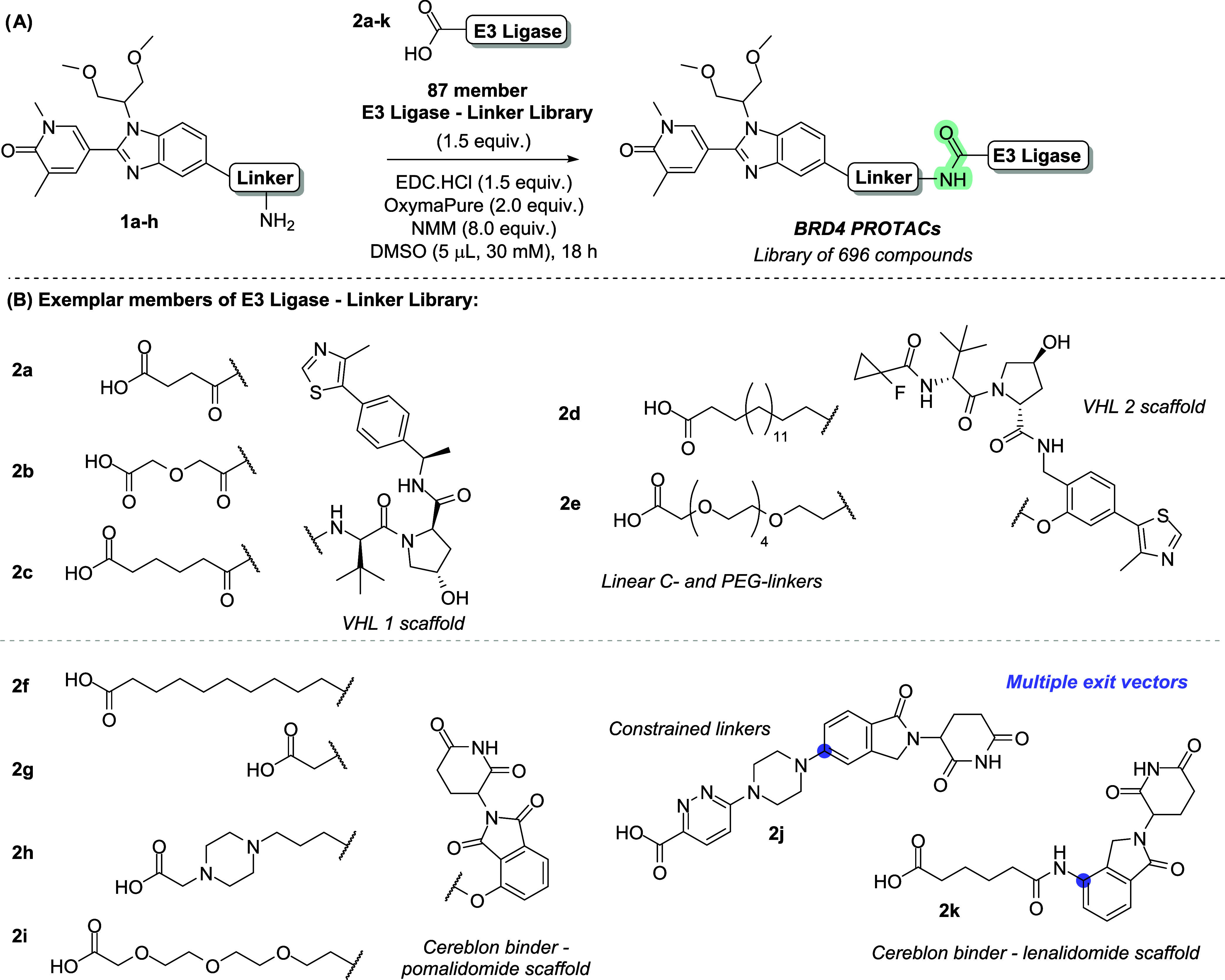
(A) Case Study for This Work—An Amide Coupling Reaction to Synthesize 696 PROTACs That Suffered Issues in Translatability between 1536-Well Plates and Batch Scale; (B) Examples of Library Members That Comprise the E3 Ligase Binder for Cereblon or VHL with Half-Linker Attached (**2a**–**k**); Different Exit Vectors Indicated in Blue

A set of eight amines were synthesized based on the BRD4 ligand I-BET469^21,32^ with a variety of short linkers attached to the benzimidazole. A library of 87 E3 ligase ligands plus linkers was compiled, selecting a diverse set of E3 ligase ligands to recruit cereblon or VHL,^33,34^ with various linear or constrained linkers attached (Scheme 1).

Convergent synthesis of PROTACs via an amide coupling of these two libraries yielded a set of 696 prospective BRD4 degraders suitable for biological evaluation.^21^ However, despite the previous success of these reaction conditions in a wide range of PROTAC projects in our laboratories, we observed almost no conversion to the desired PROTAC set when trifluoroacetic acid (TFA) salts of the amine starting materials were used. A range of factors were subsequently found to be crucial in determining reaction success despite the equivalent conditions in batch scale reactions remaining unaffected.

All reactions were assessed by LCMS, taking an aliquot from the 1536-well plate and recording the percentage of each product. For larger libraries, PyParse, an automated analysis tool,^35^ was used to assess the LCMS profiles. The library success rate was defined as the proportion of reactions that resulted in full conversion from the starting material. It is important to note that since a peak corresponding to OxymaPure is present in the LCMS trace for all reactions, product peak areas of 50–60% typically correspond to full conversion from their respective starting materials.

### Choice of Ammonium Salt Counterion or Free Base

**Figure 1:**
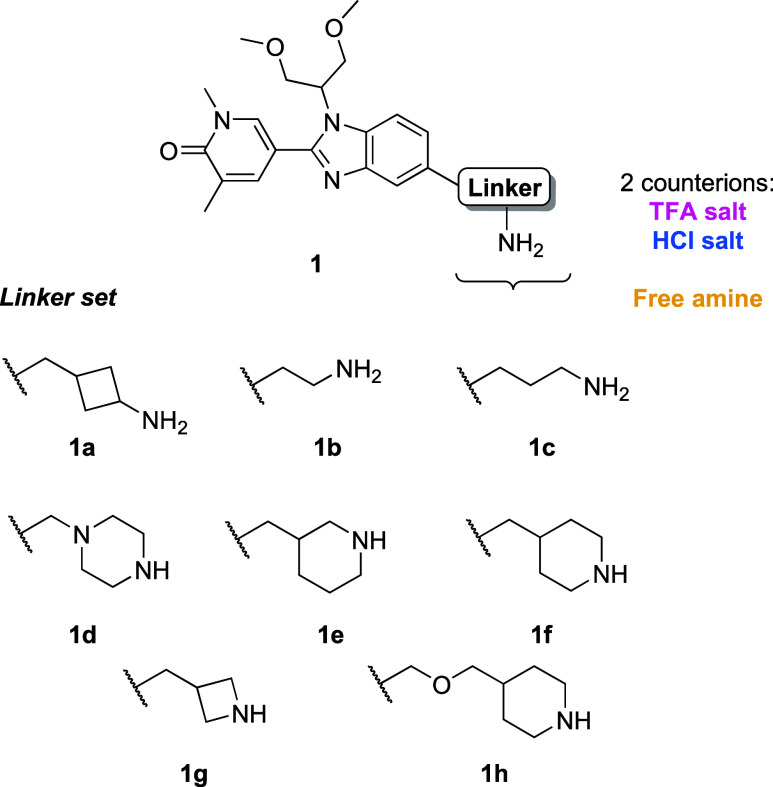
Structures of compound **1a**–**h** consisting of the BRD4 ligand I-BET469 with a series of eight short linkers attached. **1a** and **1b** will be primarily used for investigations carried out in this work either as a TFA salt (pink), HCl salt (blue), or free amine (yellow); consistent coloring is used throughout to indicate amine counterion.

**Figure 2:**
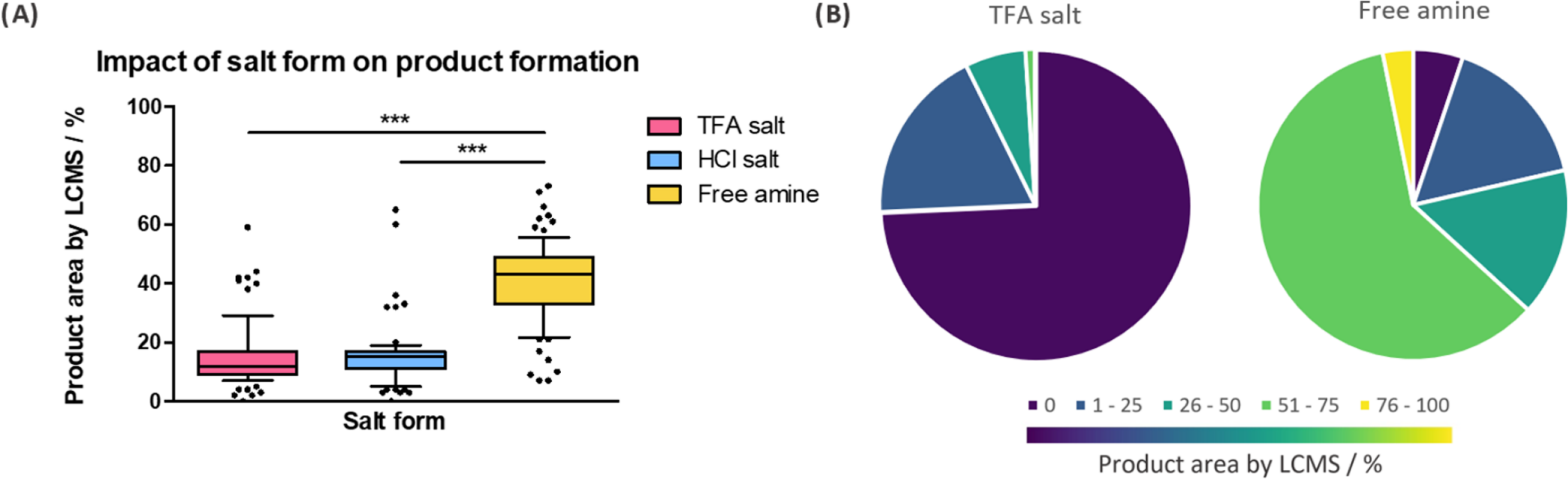
(A) Box plots to indicate reaction success rates for amine **1b** bearing two different counterions (TFA salt in pink, HCl salt in blue) and the free base/no counterion (yellow) with a library of 87 carboxylic acids. Tukey multiple comparisons test indicated a statistically significant difference between TFA and HCl salts with the free amine (***P ≤ 0.001); (B) Pie charts indicating breakdown of reaction success rate for a combinatorial library between eight amines (either with TFA counterion or as free base) and a library of 87 carboxylic acids.

Our amine library was prepared by Boc-deprotection using TFA or HCl followed by concentration of the reaction mixture to yield the corresponding salt or by passing the ammonium salts through a strong cation exchange (SCX) cartridge to yield the free amines (Figure 1). When first attempting a reaction between amines **1a**–**h** and the 87-member carboxylic acid library, the reactions employing the ammonium TFA salt resulted in poor conversion to the desired product. Analysis of the 696-member reaction set showed that 74% of the reactions contained no desired PROTAC product, and only 1% of the reactions yielded product areas of 51–75% (Figure 2B). Comparison with the free amine set revealed a stark difference in reactivity: 63% of reactions had product areas of 51% or more, and a further 15% of reactions had areas of 26–50%, which would be deemed sufficient for D2B testing in biological assays. Analysis of primary versus secondary amines in the set showed high conversion to the desired products regardless of the type of amine used (Figure S1).

To investigate this phenomenon, we compared the free amine and the salts of amine **1b**. Results revealed a statistically significant difference in product formation for the respective libraries (Figure 2A). It was initially hypothesized that the poor conversions to the desired products were due to the water content within the reactions, which may be higher for the hygroscopic TFA and HCl salts, although moisture is typically avoided by conducting 1536-well plate experiments in a glovebox.

### Coupling Reagent Quantity Impacts Reaction Outcome

**Figure 3:**
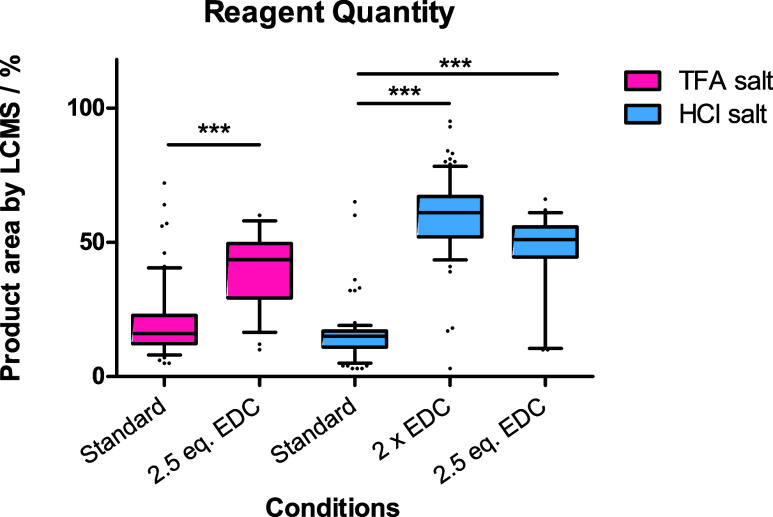
Box plots indicating reaction success rates for amine **1a** (TFA salt) or **1b** (HCl salt) with a library of carboxylic acids. Tukey multiple comparisons test indicated a statistically significant difference between reagent quantities with both TFA and HCl salts (***P ≤ 0.001). Standard = 1.5 equiv of EDC dosed first (64 or 87 monomers); 2.5 eq. EDC = 2.5 equiv of EDC dosed first (32 monomers); 2× EDC = 1.5 equiv of EDC dosed once first and again with NMM after 24 h (87 monomers).

To investigate the influence of the coupling reagent used in the reactions, two changes to the protocol were trialed. For TFA and HCl salts of amines **1a** or **1b**, an additional equivalent of EDC at the start of the reaction (to give 2.5 equiv total) was sufficient to push the majority of reactions to full conversion, with a difference in product formation observed by LCMS (Figure 3). When a reaction set with HCl salts was unsuccessful, a second addition of 1.5 equiv EDC and 8 equiv NMM after 24 h was added to the mixture. This resulted in the majority of the reactions reaching full conversion. However, a second addition of 8 equiv of the base without EDC did not improve conversion.

The requirement for additional EDC suggests that the first equivalent of EDC is consumed and leads to an unproductive pathway prior to the desired reaction, a commonly reported issue with the use of carbodiimide coupling reagents.^36−40^ While the trifluoroacetate counterion might be interfering with the reaction, EDC is dosed as a HCl salt so it is unclear how the chloride counterion could also affect reactivity. We hypothesize that this phenomenon is likely due to a mass transfer issue^41−44^ and by dosing additional EDC, either at the start or in a second dose, the interaction between reacting components is improved.

### Order of Reagent Addition Affects Conversion

Standard procedure in our laboratories is to dose out reagents in the order: amine and acid, EDC, OxymaPure then NMM. As the base is known to be more volatile than the DMSO stock solutions of the other reagents, adding NMM last prevents the risk of evaporation while on the source plate prior to dosing.

**Figure 4:**
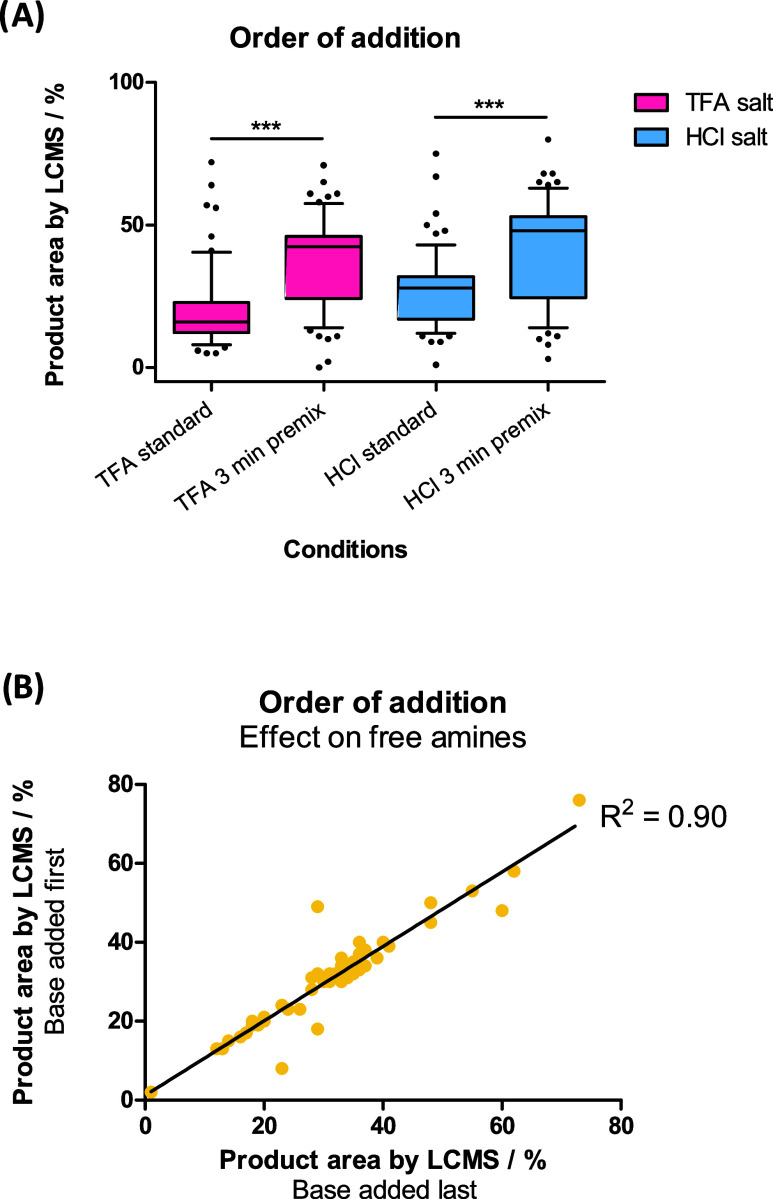
(A) Box plots indicating reaction success rates for amine **1b** bearing two different counterions (TFA salt in pink and HCl salt in blue) with a library of 64 carboxylic acids. Reactions were either dosed with base first in “3 min premix” conditions (i.e., amine, acid, NMM, 3 min pause, EDC, OxymaPure) or last in “standard” conditions (i.e., amine, acid, EDC, OxymaPure, 3 min pause, NMM). Tukey multiple comparisons test indicated a statistically significant difference between base dosing first and last with both TFA and HCl salts (***P ≤ 0.001); (B) No impact was observed for different orders of base addition with the free amine. *R*^2^ = 0.90 for run 1 versus run 2, indicating high reproducibility between runs.

It was hypothesized that “premixing” the base and amine by dosing these reagents first and leaving them to stand would improve conversion. Dosing out the amine, acid, and base followed by a 3 min pause before adding EDC and OxymaPure had a profound effect on reaction success. Although the reagents were left to stand and thus were not formally “mixed”, the library success rate for TFA and HCl salt starting materials was significantly higher when premixing these reagents for 3 min (Figure 4A).

No difference was observed when premixing with base in the cases where free amine starting materials were used, with a correlation of R^2^ = 0.9 between runs. This observation highlighted that the variation in reaction conversion is due to the intrinsic reactivity of the acids employed in the library, as opposed to variation in dosing.

### Mixing Protocol Improves Conversion

Considering that these issues were only observed on the nanoscale, we surmised that the lack of diffusion between layers of starting materials could contribute to poor conversion. 1536-well plates have a limited capacity that eliminates the option for stirrer bars, therefore reactions rely on microfluidic mixing.^11^

**Figure 5:**
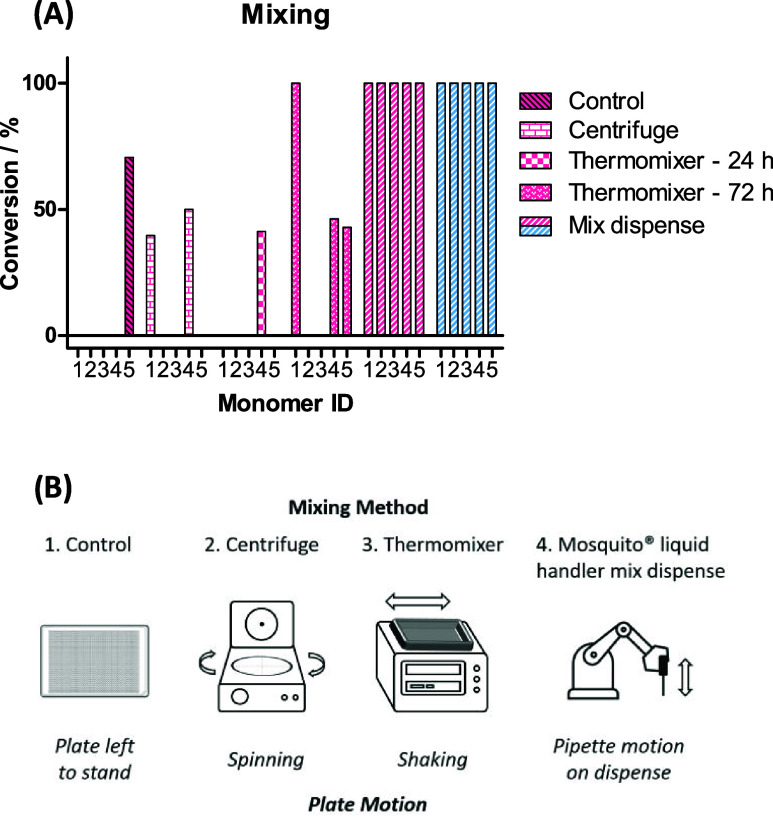
(A) Bar chart showing reaction conversion (product formation relative to amine starting material **1b**) with a set of five carboxylic acids **2a**–**e** (corresponding to entries 1–5, respectively). Reactions employing TFA salts are colored in pink and HCl salts in blue. (B) A range of different methods of mixing trialed included centrifuging the plate once dosed, shaking at 300 rpm on the thermomixer with light heating to 40 °C, leaving the plate to stand, and mixing the reaction mixtures after each reagent was dispensed.

**Scheme 2:**
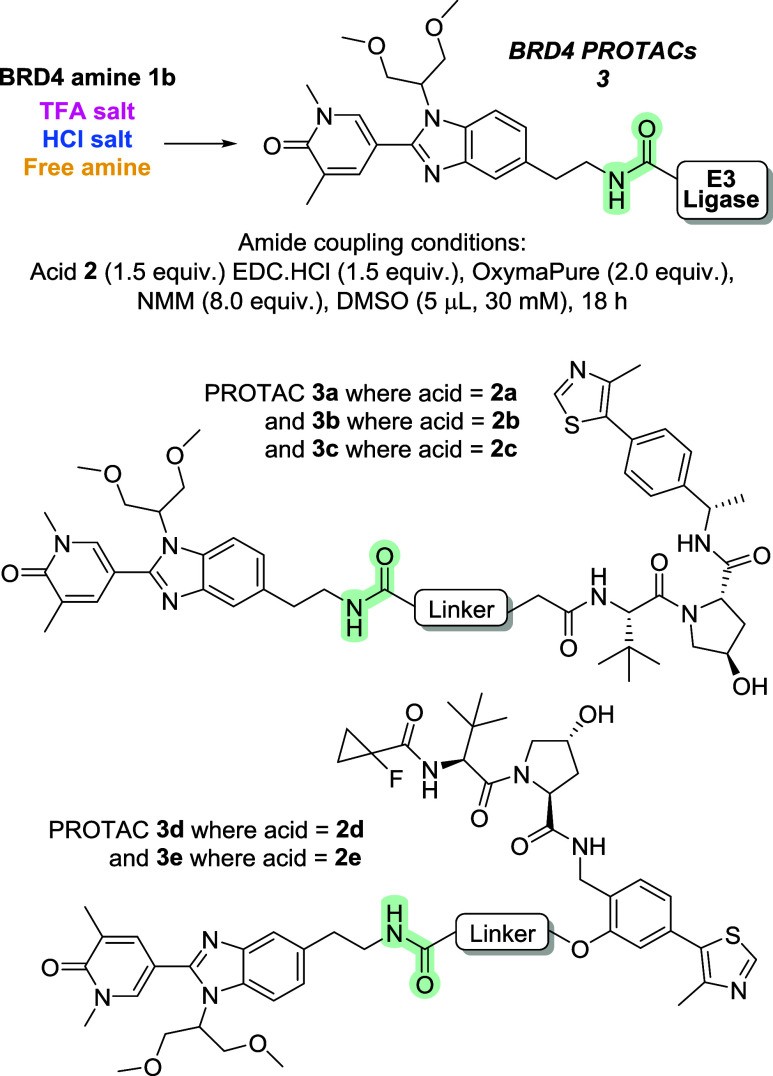
PROTACs **3a**–**e** Represent a Subset of the Library Which Were Used for Investigations into Mixing Protocol, Solvent Choice, and Resynthesis in Batch

A range of mixing protocols were assessed with a set of 5 amide coupling reactions using the TFA salt of amine **1b** and five acid monomer examples **2a**–**e** to synthesize PROTACs **3a**–**e** (Scheme 2). First, a control plate was prepared where the plate was simply left to stand after reaction dosing by a mosquito liquid handler (Figure 5B details the mixing protocols trialed). Only one monomer in the control experiment showed any conversion to the desired product (Figure 5A).

Standard practice in our laboratories was to dispense reagents using a mosquito liquid handler and then centrifuge the plate for approximately 10 s to ensure all reagents are removed from the sides of the well in advance of sealing the plate and to mix the reactions by pulling the solvent and reagents into the bottom of the wells. However, in this case, the impact of centrifuging the reaction plate for 10 s after reagent dispensing was minimal, with two examples reaching partial conversion to product.

On batch scale, heating reactions typically aid in reaction conversion. In order to mimic the use of a stirrer hot plate, a reaction plate containing the same five reaction mixtures was heated to 40 °C and shaken on a thermomixer at 300 rpm, with aliquots taken for LCMS at 24 and 72 h time points. This method improved conversion, for monomer 4 after 24 h, as well as monomers 1 and 5 after 72 h. However, these improvements were still limited to a subset of monomers and only resulted in full conversion to product in one of the examples.

The mosquito liquid handler offers a wide range of functionalities for setting up nanoscale chemistry and offers the ability to mix aliquots on either aspirating or dispensing procedures. A 3-fold mix protocol was added to each reagent dispense action with a height adjustment to aspirate from the well base and dispense 1.5 mm above the well base, i.e., approximately halfway into the core of the reaction mix, ensuring that the starting materials were well mixed. This method was highly effective in the case of both TFA and HCl salts of the starting amine, resulting in full conversion to the desired products.

The impact of these different mixing protocols on the reaction conversion highlights the subtleties of the microfluidics and indicates that mixtures may not be as homogeneous as expected without the use of mix dispense procedures. Plate centrifugation and shaking on a thermomixer were only weakly effective at facilitating reactions in 1536-well plates. These findings highlight the importance of mass transfer when carrying out reactions in several microliters, an area that has been more thoroughly investigated in the fields of flow chemistry^41^ and nanoparticle synthesis.^45^

### Different Trends Observed when Varying the Solvent

Given the results from the mixing protocol experiments indicated that mass transfer played a key role in the success of reactions in 1536-well plates, the role of solvent choice was investigated to determine whether factors such as viscosity may be significant in determining nanoscale reaction success.

**Figure 6:**
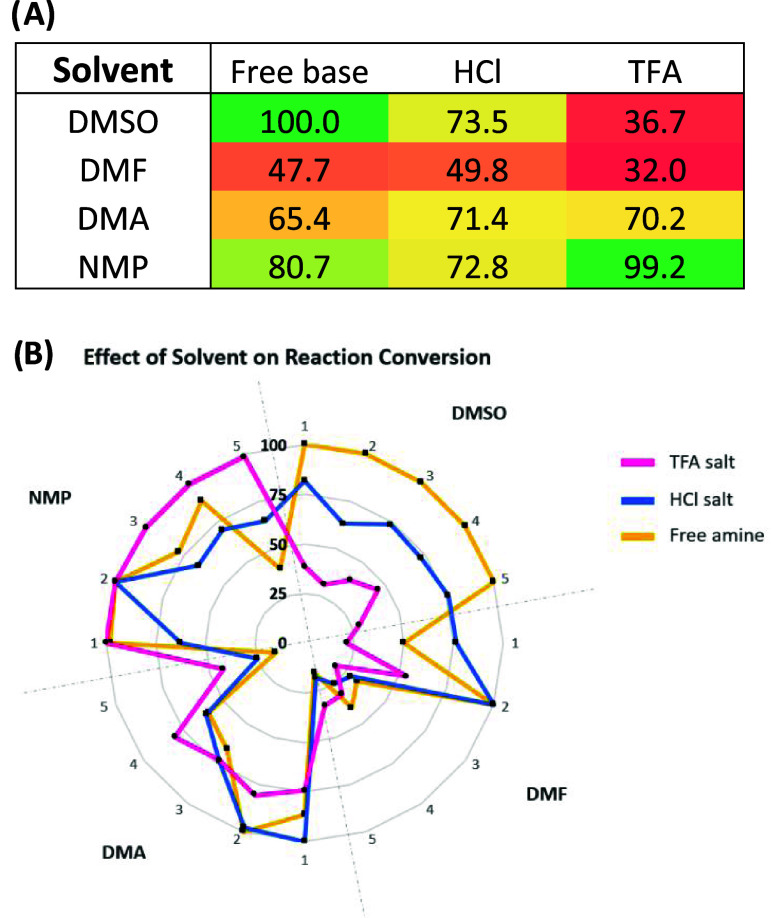
(A) Summary of the data presented in radar plot, values represent average % reaction conversion across 5 examples **3a**–**e** and colored red to green based on success rate. (B) Radar plot to show the effect of solvent choice on % reaction conversion (product formation relative to amine starting material) with three different salt forms of amine starting material **1b** (TFA salt in pink, HCl salt in blue, and free base/no counterion in yellow).

Three high-boiling solvents, NMP, DMA, and DMF, were selected due to their compatibility with the glovebox setup required for plate-based protocols. While these three solvents did not demonstrate such a significant solvent effect based on amine counterion as DMSO, substantial differences in product formation were observed as the solvent was varied (Figure 6).

NMP was identified as the optimal solvent choice for all three salt forms of amine **1b**, with reaction conversions between 72 and 99% for the three salt forms. Reactions in DMA provided moderate to good conversion, but with lower overall success rates and no reactions proceeding as smoothly as in NMP. Reactions in DMF typically gave poor conversion to the desired PROTAC, but this could be attributed to the sparing solubility of EDC in DMF. This required the coupling agent to be dosed as a suspension, which is not ideal for liquid handling on the required scale.

The reaction success rate in the nanoscale did not appear to correlate with the solvent viscosity. Reactions were typically most successful in NMP (viscosity = 1.67 cP) or DMA (2.14 cP) and least successful in DMF (0.92 cP) or DMSO (2.24 cP), but it would be expected that less viscous solvents would facilitate improved mixing rather than hindering it.

### Resynthesis in Batch Highlights Discrepancy between Plate and Vial Scale

To confirm that these findings were specific to working in 1536-well plates, a set of PROTAC examples **3a**–**e** were resynthesized on a batch scale in microwave vials with magnetic stirrer bars. The reactions were dosed using Gilson pipettes with all reagents made up in the same stock concentrations as on nanoscale, with the same order of addition. Conversion was compared for 1536-well plate versus batch scale (310-fold larger) for a set of 10 examples (Table 1).

**Table 1:**
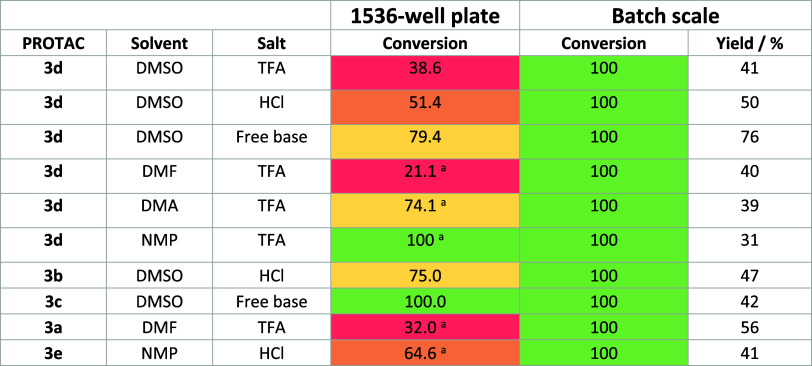
Conversion = Product/Product + Amine; Batch Scale Performed on 46.4 μmol of Amine **2a**–**e** (310-Fold Increase from 1536-Well Plate)^b^

a Mean calculated from *N* = 2.

b Yields were obtained after preparative HPLC purification.

Examples carried out in DMSO, with TFA and HCl salts, or free amine show that despite the difference in salt form having a significant impact on conversion in plate, the conversion in batch scale was 100% in all cases, corresponding to PROTAC yields over 40% after preparative HPLC purification. Further examples were carried out to compare the impacts of solvent in plate versus batch, using DMF, DMA, and NMP. These conditions also provided significantly improved conversion in batch and yields similar to those of other examples. Lastly, five different VHL ligand-linker carboxylic acids were used to synthesize five unique PROTACs **3a**–**e**. Conversion values of 100% are observed across the set of structurally diverse acids, providing evidence that this is a plate effect rather than intrinsic reactivity.

These results demonstrate significant differences in conversion between results in plate and batch, as well as highlighting the importance of the factors investigated to chemists working in nanoscale chemistry protocols, as good reproducibility between plate and batch is not always guaranteed.

## Conclusions

A nanoscale amide coupling reaction to synthesize large libraries of PROTACs in 1536-well plates was used as a case study to investigate a range of factors for their role in the success rate of ultrahigh-throughput chemistry. The use of ammonium salts rather than free amines had an adverse influence on the reaction success rate.

The quantity and number of doses of coupling reagent, order of addition of the base to the reaction, and mixing method were all variables that influenced the conversion to PROTAC products. We therefore recommend that these variables be surveyed when undertaking plate-based reaction optimization or D2B. The choice of solvent was also crucial to reaction success, despite solution dosing of reagents on batch scale not being influenced by solvent.

Our results indicate that mass transfer is crucial in consideration of the reaction success rate on 1536-well plates, and this can be adjusted by a range of methods. These findings may vary between transformations and the reaction scaffold. We recommend that these factors be considered when setting up plate-based nanoscale chemistry. This will be crucial for downstream applications, especially when inputting negative data into machine learning models, which may be the result of engineering controls for working on the nanoscale rather than inherent reactivity. Further work is required to thoroughly understand the translation between 1536-well plates and batch, and the authors encourage others to present their reproducibility challenges from working on the nanoscale.

## General Experimental Section

Solvents and reagents were purchased from commercial suppliers and used as received. If they were not commercially available, compounds were prepared according to the literature unless stated otherwise. Reactions were carried out under nitrogen and stirred using a magnetic stirrer hot plate unless stated otherwise. Reactions using the glovebox were carried out in an MBraun MB-200B glovebox with an inert N_2_ atmosphere. No unexpected or unusually high safety hazards were encountered.

### Data Analysis

Box plots represent all data points within a library (number of members per library indicated in the figure captions) with the central line indicating median, box limits at 25th and 75th percentiles, whiskers at the 10th and 90th percentiles, and individual dots to indicate data points beyond these percentiles. Analysis of the statistical significance was carried out using one-way analysis of variance (ANOVA) with a Tukey multiple comparisons test for pairwise comparison between each data set. Symbols are provided on each plot to indicate the level of statistical significance: *P ≤ 0.05, **P ≤ 0.01, ***P ≤ 0.001.

### Materials, Reagents, and Analytical

NMR spectra were recorded at ambient temperature using standard pulse methods on a Bruker AV-400 instrument at 400 MHz, a Bruker AV4 at 700 MHz, or a Bruker AVIIIHD at 600 MHz. Chemical shifts (δ) are reported in parts per million (ppm) and are reported as observed; several PROTACs have peak duplication due to rotamers. Peak assignments were chosen based on chemical shifts, integrations, and coupling constants, considering 2D analyses where necessary or the following solvent peaks: CDCl_3_ (^1^H = 7.27 ppm), DMSO-*d*_6_ (^1^H = 2.50 ppm), CD_3_OD (^1^H = 4.87 and 3.31 ppm). Coupling constants are quoted to the nearest 0.1 Hz and multiplicities are given by the following abbreviations and combinations thereof: s (singlet), d (doublet), t (triplet), q (quartet), quin (quintet), sxt (sextet), m (multiplet), and br. (broad).

LCMS analysis was carried out on a Waters Acquity UPLC instrument equipped with a BEH column (50 mm × 2.2 mm, 1.7 μm packing diameter) and Waters micromass ZQ MS using alternate-scan positive and negative electrospray. Analytes were detected as a summed UV wavelength of 210–350 nm with purity recorded from the total absorbance chromatogram (TAC). Two liquid-phase methods were used:

Formic −40 °C, 1 mL/min flow rate. Gradient elution with the mobile phases consisted of (A) water containing 0.1% (v/v) formic acid and (B) acetonitrile containing 0.1% formic acid. Gradient conditions were initially 1% B, increasing linearly to 97% B over 1.5 min, remaining at 97% for 0.1 min, and then increasing to 100% B over 0.1 min.

High pH (HpH): 40 °C, 1 mL/min flow rate. Gradient elution with the mobile phases was as follows: (A) 10 mM aqueous ammonium bicarbonate solution, adjusted to pH 10 with 0.88 M aqueous ammonia and (B) acetonitrile. Gradient conditions were initially 1% B, increasing linearly to 97% B over 1.5 min, remaining at 97% B for 0.4 min, and then increasing to 100% B over 0.1 min.

HPLC purification was conducted on an ACCQPrep H125 instrument with an XSelect CSH C18 column (150 mm × 30 mm internal diameter, 5 μm packing diameter) at ambient temperature, eluting with ammonium bicarbonate or formic acid aqueous solutions with acetonitrile using an appropriate elution gradient determined by LCMS analysis.

For low-pH methods, the solvents employed were A: 0.1% v/v solution of formic acid in water; B: 0.1% v/v solution of formic acid in acetonitrile. For high-pH methods, the solvents employed were A: 10 mM ammonium bicarbonate in water adjusted to pH 10 with ammonia solution; B: acetonitrile. Appropriate elution gradients were determined by the following LCMS analysis: Method A: Eluting with 0–30% gradient; Method B: Eluting with 10–40% gradient; Method C: Eluting with 20–50% gradient; Method D: Eluting with 30–60% gradient; Method E: Eluting with 40–70% gradient; Method F: Eluting with 50–80% gradient.

HRMS analysis was carried out on a Waters XEVO G2-XS quadrupole time-of-flight instrument using positive electrospray ionization.

### Synthesis

#### Standard Procedure

Nanoscale reaction mixtures are dispensed into 1536-well microtiter plates using a mosquito liquid handler in the glovebox under an inert N_2_ atmosphere. Reactions are carried out in 5 μL of DMSO at a concentration of 30 mM. Room-temperature reactions are left overnight in a sealed plate without stirring or agitation. Heated reactions are sealed and placed in a thermomixer overnight at the desired temperature with shaking at 300 rpm.

After 18–24 h, an aliquot of 0.5 μL of reaction mixture is taken and diluted with 39.5 μL of acetic acid in acetonitrile for LCMS analysis on 2 min formic method. PROTAC purity is determined by % area in the LCMS UV trace and thus is not the same as conversion or product concentration. PyParse is used to automate the analysis process, with the raw data file input and spreadsheet of the LCMS purity output.

The following reagents are used for the amide coupling transformation (in this order of addition unless otherwise stated): 0.15 μmol of amine made up to 1.5 μL with DMSO per well (0.1 M, 1 equiv), 0.15 μmol of acid made up to 1.5 μL with DMSO per well (0.1 M, 1 equiv), 0.225 μmol of EDC·HCl made up to 1.28 μL with DMSO per well (0.176 M, 1.5 equiv), 0.3 μmol of OxymaPure made up to 0.589 μL with DMSO per well (0.509 M, 2 equiv), 131 nL neat NMM per well (8 equiv/1.2 μmol).

Adjustments to the standard procedure to investigate each factor discussed in this article are detailed below.

#### Salt Forms

Reactions were prepared according to the standard procedure, with alteration in the choice of amine starting material.

#### Reagent Quantity

“Standard” conditions indicate the use of 1.5 equiv of EDC as detailed in the standard procedure above.

Conditions indicating “2.5 equiv EDC” were prepared with an additional equivalent of EDC at the start of the reaction (to give 2.5 equiv total).

Conditions indicating “2× EDC” represent the setup of a standard reaction plate, followed by a second addition of 1.5 equiv of EDC after 24 h.

#### Order of Addition

“Standard” conditions indicate the addition of NMM last to the reaction mixtures as detailed in the standard procedure above.

“3 min premix” conditions indicate the addition of NMM after the acid and amine, with the reactions left to stand for 3 min prior to the addition of EDC and OxymaPure.

#### Mixing Protocol

For “centrifuge” examples, the reaction mixtures were dosed according to standard procedure then placed into a benchtop plate centrifuge and spun for approximately 10 s, then left to stand for the reaction duration.

For “thermomixer” examples, reaction mixtures were dosed according to standard procedure then heated to 40 °C and shaken on a thermomixer at 300 rpm for either 24 or 72 h.

For mix dispense examples, a 3x mix protocol was added to each reagent dispense action on the mosquito with a height adjustment to aspirate from the well base and dispense 1.5 mm above the well base and then left to stand for the reaction duration.

#### Solvent Choice

Reactions were prepared according to standard procedure, with the exception of preparing all stock solutions in an alternative solvent to DMSO. Solvents used were DMA, DMF, and NMP.

#### Batch Scale Synthesis

For synthesis on batch scale, characterization data, and compound spectra, see the Supporting Information.

## Data Availability

The data underlying this study are available in the published article and its Supporting Information.

## Supporting Information Available

Batch scale synthesis, characterization data, compound spectra, and data tables to support the plots shown in the publication (PDF)

## Abbreviations

AI: artificial intelligence
D2B: direct-to-biology
DMA: *N,N*-dimethylacetamide
DMF: *N,N-*dimethylformamide
DMSO: dimethyl sulfoxide
EDC: 1-ethyl-3-(3-(dimethylamino)propyl)carbodiimide
TFA: trifluoroacetic acid
HTE: high-throughput experimentation
HTS: high-throughput screening
LCMS: liquid chromatography–mass spectrometry
NMM: 4-methylmorpholine
NMP: 1-methyl-2-pyrrolidinone
OxymaPure: ethyl cyano(hydroxyimino)acetate
PROTAC: proteolysis targeting chimera
TAC: total absorbance chromatogram
UV: ultraviolet
