# Supplementary material for: Factors to Consider for Synthesis in 1536-Well Plates—An Amide Coupling Case Study for PROTAC Synthesis
Source: J Org Chem. 2025 Feb 3;90(6):2192–200. doi: 10.1021/acs.joc.4c02456 (PMC11833857; doi:10.1021/acs.joc.4c02456)
Supplement: Supplementary file 1 — jo4c02456_si_001.pdf [file jo4c02456_si_001.pdf]

Supporting Information

Factors to Consider for Synthesis in 1536-Well Plates – An Amide Coupling Case Study for PROTAC Synthesis

Rebecca Stevens[1,2]*, Harry E. P. Palmer[1,2], Afjal H. Miah[1], Glenn A. Burley[2]*

1) Modality Platform Technologies, GSK, Stevenage, SG1 2NY, UK

2) Department of Pure and Applied Chemistry, University of Strathclyde, Glasgow, G1 1BX

*Corresponding authors: rebecca.8.stevens@gsk.com and glenn.a.burley@strath.ac.uk

Contents

Additional Data Analysis S2

Starting Material Synthesis S3

Batch Scale Synthesis S5

NMR Spectra S11

Batch Scale Resynthesis S11

Compound 3a – ^{1}H NMR (600 MHz, DMSO-d_6) S11

Compound 3a – ^{13}C {^{1}H} NMR (151 MHz, DMSO-d_6) S12

Compound 3b – ^{1}H NMR (600 MHz, DMSO-d_6) S12

Compound 3b – ^{13}C {^{1}H} NMR (151 MHz, DMSO-d_6) S13

Compound 3c – ^{1}H NMR (600 MHz, DMSO-d_6) S13

Compound 3c – ^{13}C {^{1}H} NMR (151 MHz, DMSO-d_6) S14

Compound 3d – ^{1}H NMR (600 MHz, DMSO-d_6) S14

Compound 3d – ^{13}C {^{1}H} NMR (151 MHz, DMSO-d_6) S15

Compound 3d – ^{19}F NMR (376 MHz, DMSO-d_6) S15

Compound 3e – ^{1}H NMR (600 MHz, DMSO-d_6) S16

Compound 3e – ^{13}C {^{1}H} NMR (151 MHz, DMSO-d_6) S16

Compound 3e – ^{19}F NMR (376 MHz, DMSO-d_6) S17

Data Tables S18

Figure 2A S18

Figure 2B S19

Figure 3 .... S30

Figure 4A S31

Figure 4B S32

Figure 5A S33

Figure 6A and 6B S34

Table 1 .... S35

References S35

Additional Data Analysis

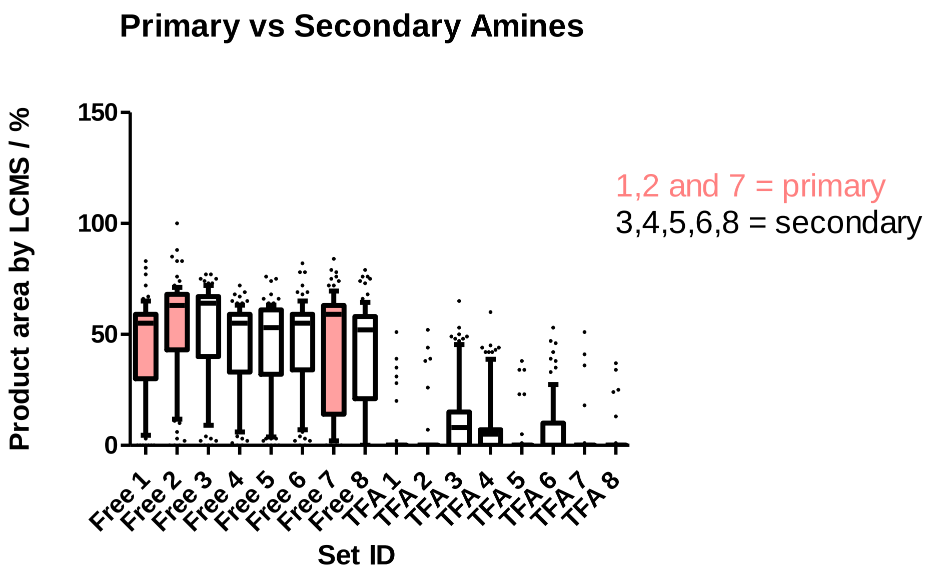

Figure S1. Analysis of reaction success rate for a combinatorial library between eight amines (either with TFA counterion or as free base) and a library of 87 carboxylic acids. Amines 1, 2 and 7 are primary with box plots coloured in pink, and amines 3,4,5,6 and 8 are secondary with no colour in the box plots.

An analysis of libraries with primary and secondary amines showed a non-statistically significant difference in percentage product formed based on amine type in the case of either the TFA counterion or free base of the amine. Minimal variation between different monomers was observed, but a significant difference between the two amine counterions was observed, as discussed further in the article main text.

Starting Material Synthesis

1a-h were prepared according to literature procedure[1] by palladium-catalysed sp^2-sp^3 cross-coupling followed by Boc-deprotection (Scheme S1). Isolation of trifluoroacetic acid salts and free amines was performed according to direct literature precedent, by deprotection with TFA to give the salts, and subsequent purification by SCX cartridge and elution with ammonia in methanol to give yield the free amines.

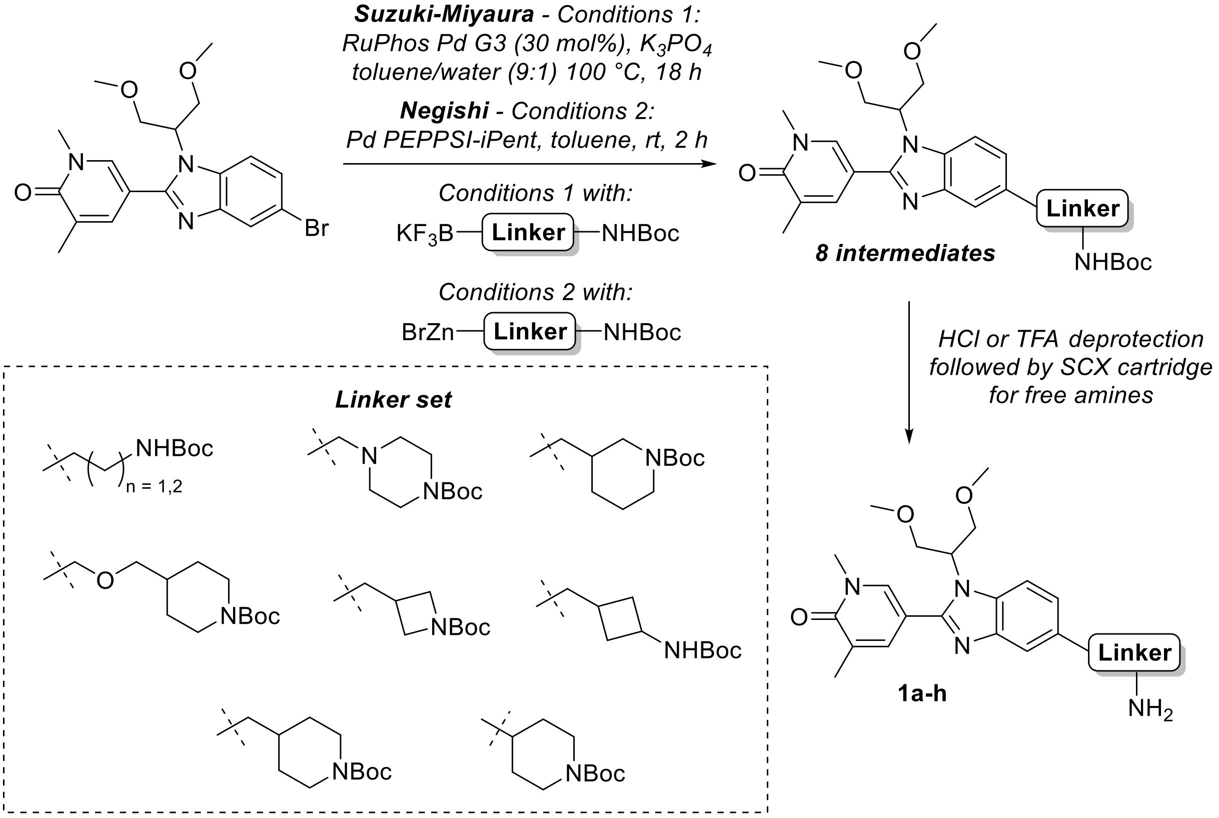

*Scheme S1. Synthesis of **1a-h** according to literature procedure.*[1]

Hydrochloric acid salts **1a** and **1b** were prepared as below; characterisation data found to be consistent with the corresponding TFA salts in the literature.[1]

5-(5-((3-Aminocyclobutyl)methyl)-1-(1,3-dimethoxypropan-2-yl)-1*H*-benzo[*d*]imidazol-2-yl)-1,3-dimethylpyridin-2(1*H*)-one; hydrochloride salt

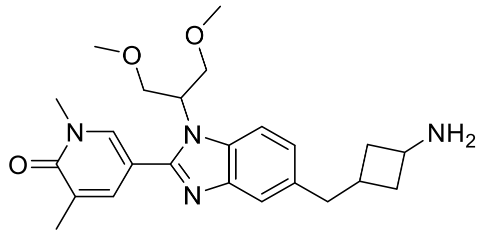

1a

To *tert*-butyl (3-((1-(1,3-dimethoxypropan-2-yl)-2-(1,5-dimethyl-6-oxo-1,6-dihydropyridin-3-yl)-1*H*-benzo[*d*]imidazol-5-yl)methyl)cyclobutyl)carbamate (89.0 mg, 1 Eq, 169.6 µmol) was added hydrochloric acid (636.1 µL, 4 M in dioxane, 15 Eq) and the reaction mixture was stirred at room temperature for 4 h, then concentrated under a stream of nitrogen to give the title product as a yellow oil (79.0 mg, 171 µmol, 96% yield).

^{1}H NMR: (400 MHz, $CDCl_3$) δ 8.89 (br s, 3H), 8.46 (d, *J* = 8.9 Hz, 1H), 7.85 (s, 1H), 7.79 – 7.72 (m, 1H), 7.72 – 7.61 (m, 1H), 7.35 – 7.28 (m, 1H), 5.09 – 4.97 (m, 1H), 4.14 – 4.03 (m, 2H), 3.93 – 3.85 (m, 2H), 3.71 (s, 3H), 3.71 – 3.66 (m, 4H), 3.32 (br d, *J* = 2.5 Hz, 6H), 3.21 – 3.15 (m, 1H), 2.93 (br d, *J* = 7.9 Hz, 1H), 2.68 – 2.58 (m, 1H), 2.51 – 2.43 (m, 1H), 2.22 – 2.18 (m, 3H); **LCMS:** t_R = 0.82 min (100% purity by TAC) using 2 min HpH method as described in general experimental, $[M+H]^+$ 425.3.

5-(5-(2-Aminoethyl)-1-(1,3-dimethoxypropan-2-yl)-1*H*-benzo[*d*]imidazol-2-yl)-1,3-dimethylpyridin-2(1*H*)-one; hydrochloride salt

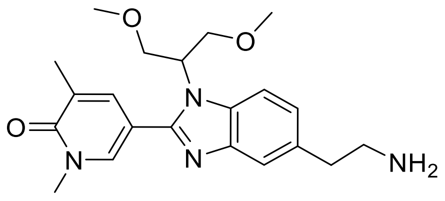

1b

To *tert*-butyl (2-(1-(1,3-dimethoxypropan-2-yl)-2-(1,5-dimethyl-6-oxo-1,6-dihydropyridin-3-yl)-1*H*-benzo[*d*]imidazol-5-yl)ethyl)carbamate (96.0 mg, 1 Eq, 198.1 µmol) was added hydrochloric acid (571.6 µL, 4 M in dioxane, 15 Eq) and the reaction mixture was stirred at room temperature for 4 h, then concentrated under a stream of nitrogen to give the title product as a colourless oil (84.0 mg, 190 µmol, 96% yield).

^{1}H NMR: (400 MHz, $CDCl_3$) δ 8.90 (br s, 3H), 8.48 (d, *J* = 2.0 Hz, 1H), 8.10 (br s, 1H), 7.81 – 7.73 (m, 2H), 7.44 – 7.37 (m, 1H), 5.00 – 4.90 (m, 1H), 4.11 – 4.01 (m, 2H), 3.88 (dd, *J* = 10.3, 4.2 Hz, 2H), 3.69 (s, 3H), 3.41 – 3.33 (m, 4H), 3.30 (s, 6H), 2.21 (s, 3H); **LCMS:** t_R = 0.71 min (100% purity by TAC) using 2 min HpH method as described in general experimental, $[M+H]^+$ 385.2.

Compounds **2** including **2a**-**k** are a library of carboxylic acids comprising 87 E3 ligase ligands with linkers attached. These compounds were synthesised according to common literature methods.[2, 3]

Batch Scale Synthesis

Ten example PROTACs were prepared in batch scale to assess the impact of scale on conversion, then compounds were purified, isolated and full characterisation was performed. All compounds were resynthesized on a 310-fold scale up from 1536-well plate synthesis. Preparation and characterisation of unique compounds **3a-e** is detailed below; remaining five examples were synthesised according to the same methods with yields and conversion data shown in Table 1.

***N*¹-(2-(1-(1,3-dimethoxypropan-2-yl)-2-(1,5-dimethyl-6-oxo-1,6-dihydropyridin-3-yl)-1*H*-benzo[*d*]imidazol-5-yl)ethyl)-*N*⁴-((*S*)-1-((2*S*,4*R*)-4-hydroxy-2-(((*S*)-1-(4-(4-methylthiazol-5-yl)phenyl)ethyl)carbamoyl)pyrrolidin-1-yl)-3,3-dimethyl-1-oxobutan-2-yl)succinamide**

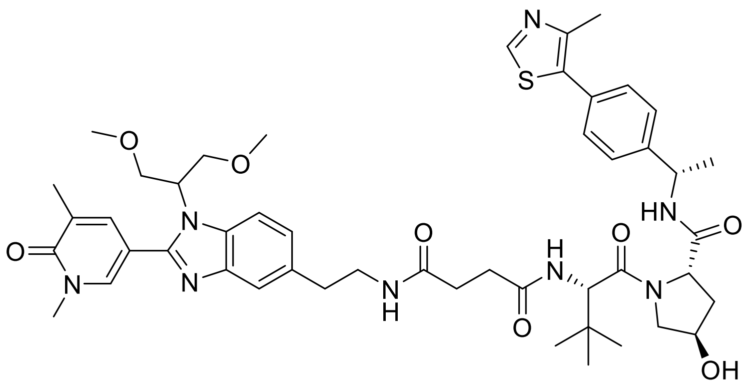

3a

To a solution of 4-(((*S*)-1-((2*S*,4*R*)-4-hydroxy-2-(((*S*)-1-(4-(4-methylthiazol-5-yl)phenyl)ethyl) carbamoyl)pyrrolidin-1-yl)-3,3-dimethyl-1-oxobutan-2-yl)amino)-4-oxobutanoic acid (37.9 mg, 464.2 µL, 0.150 molar, 1.50 Eq, 69.63 µmol) in DMF was added a solution of 5-(5-(2-aminoethyl)-1-(1,3-dimethoxypropan-2-yl)-1*H*-benzo[*d*]imidazol-2-yl)-1,3-dimethylpyridin-2(1*H*)-one, trifluoroacetic acid salt (23.1mg, 464.2 µL, 0.100 molar, 1 Eq, 46.419 µmol) in DMF, EDC (13.3 mg, 395.6 µL, 0.176 molar, 1.5 Eq, 69.63 µmol) in DMF, cyanic (*E*)-2-(hydroxyimino)butanoic anhydride (13.2 mg, 182.4 µL, 0.509 molar, 2 Eq, 92.838 µmol) in DMF and *N*-methylmorpholine (37.6 mg, 40.8 µL, 8 Eq, 371.352 µmol) and the reaction mixture was stirred at room temperature for 18 h in a sealed microwave vial. The reaction mixture was purified directly using Accqprep (HpH Method C) and the relevant fractions combined and concentrated *in vacuo* to give the title product as a colourless gum (24.8 mg, 26 µmol, 56% yield).

^{1}H NMR: (600 MHz, DMSO-d_6) δ 8.98 (s, 1H), 8.39 (d, *J* = 7.6 Hz, 1H), 8.04 (d, *J* = 2.5 Hz, 1H), 7.95 (t, *J* = 5.6 Hz, 1H), 7.86 (d, *J* = 9.4 Hz, 1H), 7.71 (d, *J* = 8.4 Hz, 1H), 7.67 (dd, *J* = 2.5, 1.1 Hz, 1H), 7.46 – 7.41 (m, 3H), 7.40 – 7.35 (m, 2H), 7.07 (dd, *J* = 8.4, 1.5 Hz, 1H), 5.12 (br s, 1H), 4.91 (quin, *J* = 7.3 Hz, 1H), 4.82 (dt, *J* = 8.7, 4.4 Hz, 1H), 4.50 (d, *J* = 9.4 Hz, 1H), 4.43 (t, *J* = 8.0 Hz, 1H), 4.28 (br s, 1H), 4.00 (dd, *J*

= 10.4, 8.9 Hz, 2H), 3.76 (dd, *J* = 10.4, 4.5 Hz, 2H), 3.65 – 3.55 (m, 2H), 3.54 (s, 4H), 3.31 – 3.27 (m, 2H), 3.17 – 3.14 (m, 6H), 2.80 (t, *J* = 7.3 Hz, 2H), 2.46 – 2.44 (m, 3H), 2.39 – 2.26 (m, 3H), 2.09 (s, 3H), 2.07 – 1.97 (m, 1H), 1.82 – 1.75 (m, 1H), 1.37 (d, *J* = 7.3 Hz, 3H), 0.96 – 0.91 (m, 9H); **^{13}C{^{1}H} NMR:** (151 MHz, DMSO-d_6) δ 171.3, 171.3, 170.6, 169.5, 161.7, 151.7, 151.5, 147.7, 144.7, 139.1, 137.3, 133.4, 132.0, 131.1, 129.7, 128.8, 127.5, 126.4, 123.2, 118.7, 112.2, 107.8, 69.7, 68.8, 58.6, 58.4, 56.9, 56.5, 56.2, 47.7, 40.8, 37.7, 37.6, 35.3, 35.2, 31.0, 30.6, 26.4, 22.4, 17.0, 16.0; **LCMS:** t_R = 0.95 min (96% purity by TAC) using 2 min HpH method as described in general experimental, $[M+H]^+$ 911.4; **HRMS (ESI-TOF) m/z:** $[M+H]^+$ Calcd for $C_{48}H_{63}N_8O_8S$ 911.4484; found 911.4468.

(2*R*,4*S*)-1-((*R*)-2-(2-(2-((2-(1-(1,3-Dimethoxypropan-2-yl)-2-(1,5-dimethyl-6-oxo-1,6-dihydropyridin-3-yl)-1*H*-benzo[*d*]imidazol-5-yl)ethyl)amino)-2-oxoethoxy)acetamido)-3,3-dimethylbutanoyl)-4-hydroxy-*N*-((*R*)-1-(4-(4-methylthiazol-5-yl)phenyl)ethyl)pyrrolidine-2-carboxamide

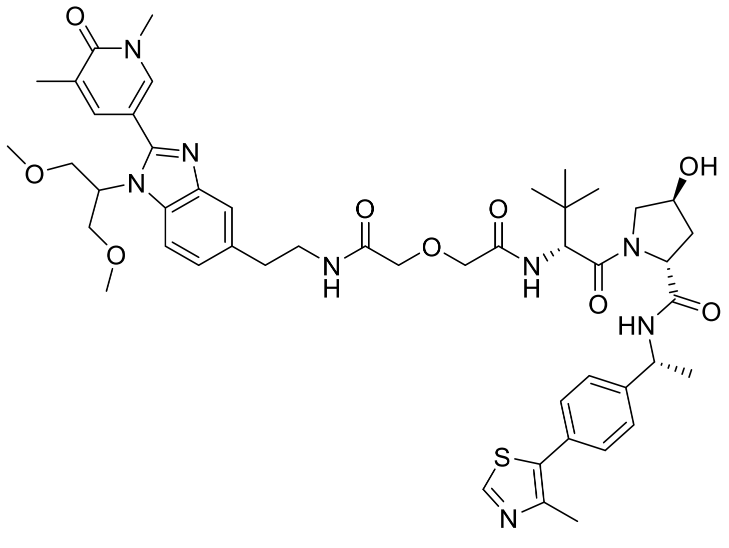

3b

To a solution of 2-(2-(((*S*)-1-((2*S*,4*R*)-4-hydroxy-2-(((*S*)-1-(4-(4-methylthiazol-5-yl)phenyl)ethyl) carbamoyl)pyrrolidin-1-yl)-3,3-dimethyl-1-oxobutan-2-yl)amino)-2-oxoethoxy)acetic acid (39.0 mg, 464.2 µL, 0.150 molar, 1.5 Eq, 69.629 µmol) in DMSO was added a solution of 5-(5-(2-aminoethyl)-1-(1,3-dimethoxypropan-2-yl)-1*H*-benzo[*d*]imidazol-2-yl)-1,3-dimethylpyridin-2(1*H*)-one, Hydrochloride (19.5 mg, 464.190 µL, 0.100 molar, 1 Eq, 46.419 µmol) in DMSO, EDC (13.3 mg, 395.6 µL, 0.176 molar, 1.5 Eq, 69.629 µmol) in DMSO, cyanic (*E*)-2-(hydroxyimino)butanoic anhydride (13.2 mg, 182.4 µL, 0.509 molar, 2 Eq, 92.838 µmol) in DMSO and *N*-methylmorpholine (37.6 mg, 40.8 µL, 8 Eq, 371.352 µmol) and the reaction mixture was stirred at room temperature for 18 h in a sealed microwave vial. The reaction mixture was purified directly using Accqprep (HpH Method C) and the relevant

fractions combined and concentrated *in vacuo* to give the title product as a colourless gum (21.2 mg, 22 µmol, 47% yield).

^{1}H NMR: (600 MHz, DMSO-d_6) δ 8.98 (s, 1H), 8.45 (d, *J* = 8.0 Hz, 1H), 8.14 (t, *J* = 5.8 Hz, 1H), 8.03 (d, *J* = 2.2 Hz, 1H), 7.75 (d, *J* = 9.4 Hz, 1H), 7.70 (d, *J* = 8.4 Hz, 1H), 7.67 (dd, *J* = 2.2, 1.1 Hz, 1H), 7.47 (d, *J* = 1.1 Hz, 1H), 7.44 – 7.41 (m, 2H), 7.36 (d, *J* = 8.4 Hz, 2H), 7.08 (dd, *J* = 8.4, 1.5 Hz, 1H), 5.15 (d, *J* = 3.6 Hz, 1H), 4.91 (quin, *J* = 7.2 Hz, 1H), 4.81 (tt, *J* = 8.9, 4.5 Hz, 1H), 4.56 (d, *J* = 9.8 Hz, 1H), 4.48 – 4.44 (m, 1H), 4.29 (br s, 1H), 4.05 – 3.96 (m, 6H), 3.76 (td, *J* = 5.4, 4.2 Hz, 2H), 3.65 – 3.57 (m, 2H), 3.53 (s, 3H), 3.42 – 3.37 (m, 2H), 3.17 – 3.14 (m, 6H), 2.86 (t, *J* = 7.4 Hz, 2H), 2.45 (s, 3H), 2.08 (s, 3H), 2.06 – 2.01 (m, 1H), 1.79 (ddd, *J* = 13.0, 8.6, 4.5 Hz, 1H), 1.36 (d, *J* = 6.9 Hz, 3H), 0.97 – 0.95 (m, 9H); **^{13}C{^{1}H} NMR:** (151 MHz, DMSO-d_6) δ 170.5, 169.0, 168.6, 168.4, 161.7, 151.8, 151.5, 147.7, 144.7, 143.3, 139.0, 137.3, 133.0, 132.1, 131.1, 129.7, 128.8, 127.4, 126.3, 123.1, 118.8, 112.2, 108.0, 70.4, 70.1, 69.7, 68.8, 58.6, 58.4, 56.8, 56.5, 56.0, 47.7, 40.4, 37.7, 37.5, 36.2, 35.6, 35.2, 26.3, 22.4, 17.0, 16.0; **LCMS:** t_R = 0.96 min (100% purity by TAC) using 2 min HpH method as described in general experimental, $[M+H]^+$ 927.4; **HRMS (ESI-TOF) m/z:** $[M+H]^+$ Calcd for $C_{48}H_{63}N_8O_9S$ 927.4434; found 927.4429.

N^1-(2-(1-(1,3-dimethoxypropan-2-yl)-2-(1,5-dimethyl-6-oxo-1,6-dihydropyridin-3-yl)-1*H*-benzo[*d*]imidazol-5-yl)ethyl)-N^6-((*R*)-1-((2*R*,4*S*)-4-hydroxy-2-(((*R*)-1-(4-(4-methylthiazol-5-yl)phenyl)ethyl)carbamoyl)pyrrolidin-1-yl)-3,3-dimethyl-1-oxobutan-2-yl)adipamide

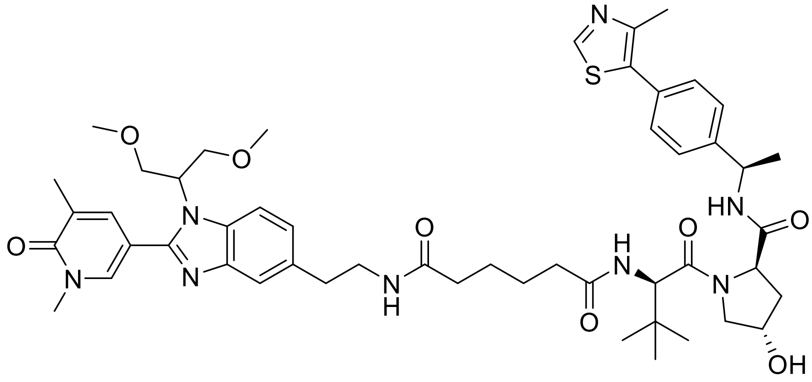

3c

To a solution of 6-(((*S*)-1-((2*S*,4*R*)-4-hydroxy-2-(((*S*)-1-(4-(4-methylthiazol-5-yl)phenyl)ethyl) carbamoyl)pyrrolidin-1-yl)-3,3-dimethyl-1-oxobutan-2-yl)amino)-6-oxohexanoic acid (39.9 mg, 464.2 µL, 0.150 molar, 1.5 Eq, 69.629 µmol) in DMSO was added a solution of 5-(5-(2-aminoethyl)-1-(1,3-dimethoxypropan-2-yl)-1*H*-benzo[*d*]imidazol-2-yl)-1,3-dimethylpyridin-2(1*H*)-one (17.8 mg, 464.2 µL,

0.100 molar, 1 Eq, 46.419 µmol) in DMSO, EDC (13.3 mg, 395.6 µL, 0.176 molar, 1.5 Eq, 69.629 µmol) in DMSO, cyanic (*E*)-2-(hydroxyimino)butanoic anhydride (13.2 mg, 182.4 µL, 0.509 molar, 2 Eq, 92.838 µmol) in DMSO and *N*-methylmorpholine (37.6 mg, 40.8 µL, 8 Eq, 371.352 µmol) and the reaction mixture was stirred at room temperature for 18 h in a sealed microwave vial. The reaction mixture was purified directly using Accqprep (HpH Method C) and the relevant fractions combined and concentrated *in vacuo* to give the title product as a colourless gum (19.4 mg, 20 µmol, 42% yield).

^{1}H NMR: (600 MHz, DMSO-d_6) δ 8.98 (s, 1H), 8.38 (d, *J* = 7.6 Hz, 1H), 8.03 (d, *J* = 2.5 Hz, 1H), 7.87 (t, *J* = 5.6 Hz, 1H), 7.79 (d, *J* = 9.1 Hz, 1H), 7.69 (d, *J* = 8.4 Hz, 1H), 7.68 – 7.66 (m, 1H), 7.44 – 7.41 (m, 3H), 7.39 – 7.36 (m, 2H), 7.06 (dd, *J* = 8.4, 1.5 Hz, 1H), 5.11 (d, *J* = 2.9 Hz, 1H), 4.92 (t, *J* = 7.3 Hz, 1H), 4.81 (tt, *J* = 8.9, 4.4 Hz, 1H), 4.52 (d, *J* = 9.1 Hz, 1H), 4.43 (t, *J* = 8.0 Hz, 1H), 4.28 (br s, 1H), 4.02 – 3.98 (m, 2H), 3.75 (dd, *J* = 10.4, 4.5 Hz, 2H), 3.64 – 3.58 (m, 2H), 3.54 – 3.53 (m, 3H), 3.32 – 3.28 (m, 2H), 3.17 – 3.15 (m, 6H), 2.80 (t, *J* = 7.3 Hz, 2H), 2.45 (s, 3H), 2.27 – 2.21 (m, 1H), 2.13 – 1.98 (m, 7H), 1.79 (ddd, *J* = 12.9, 8.5, 4.7 Hz, 1H), 1.50 – 1.42 (m, 4H), 1.37 (d, *J* = 6.9 Hz, 3H), 0.94 (s, 9H); **^{13}C{^{1}H} NMR:** (151 MHz, DMSO-d_6) δ 171.9, 171.9, 170.6, 169.6, 161.7, 151.7, 151.5, 147.7, 144.6, 143.3, 139.0, 137.3, 133.2, 132.1, 131.1, 129.7, 128.8, 127.5, 126.4, 123.1, 118.8, 112.1, 108.0, 69.7, 68.8, 58.6, 58.4, 56.8, 56.4, 56.3, 47.7, 40.7, 37.7, 37.5, 36.0, 35.3, 35.2, 35.2, 34.7, 26.5, 25.1, 25.0, 22.4, 17.0, 16.0; **LCMS:** t_R = 0.96 min (99% purity by TAC) using 2 min HpH method as described in general experimental, $[M+H]^+$ 939.5; **HRMS (ESI-TOF) m/z:** $[M+H]^+$ Calcd for $C_{50}H_{67}N_8O_8S$ 939.4797, found 939.4810.

(2*S*,4*R*)-*N*-(2-((16-((2-(1-(1,3-Dimethoxypropan-2-yl)-2-(1,5-dimethyl-6-oxo-1,6-dihydropyridin-3-yl)-1*H*-benzo[*d*]imidazol-5-yl)ethyl)amino)-16-oxohexadecyl)oxy)-4-(4-methylthiazol-5-yl)benzyl)-1-((*S*)-2-(1-fluorocyclopropane-1-carboxamido)-3,3-dimethylbutanoyl)-4-hydroxypyrrolidine-2-carboxamide

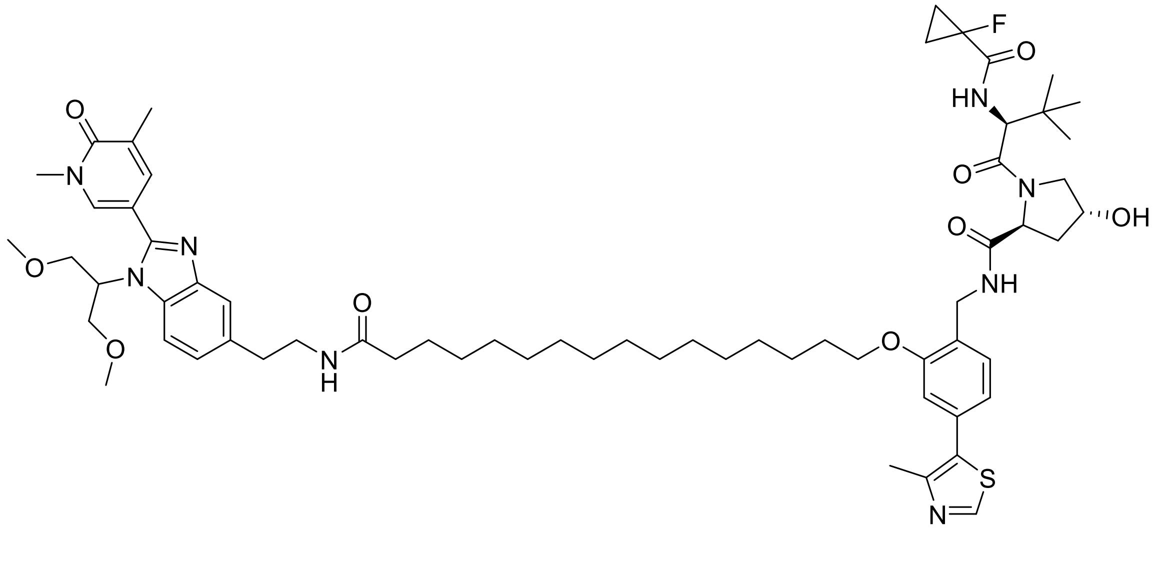

3d

To a solution of 16-(2-(((2*S*,4*R*)-1-((*S*)-2-(1-fluorocyclopropane-1-carboxamido)-3,3-dimethylbutanoyl) -4-hydroxypyrrolidine-2-carboxamido)methyl)-5-(4-methylthiazol-5-yl)phenoxy)hexadecanoic acid (54.8 mg, 464.2 µL, 0.150 molar, 1.5 Eq, 69.629 µmol) in DMSO was added a solution of 5-(5-(2-aminoethyl)-1-(1,3-dimethoxypropan-2-yl)-1*H*-benzo[*d*]imidazol-2-yl)-1,3-dimethylpyridin-2(1*H*)-one, trifluoroacetic acid salt (23.1 mg, 464.2 µL, 0.100 molar, 1 Eq, 46.419 µmol) in DMSO, EDC (13.3 mg, 395.6 µL, 0.176 molar, 1.5 Eq, 69.629 µmol) in DMSO, cyanic (*E*)-2-(hydroxyimino)butanoic anhydride (13.2 mg, 182.4 µL, 0.509 molar, 2 Eq, 92.838 µmol) in DMSO and *N*-methylmorpholine (37.6 mg, 40.8 µL, 8 Eq, 371.352 µmol) and the reaction mixture was stirred at room temperature for 18 h in a sealed microwave vial. LCMS showed full conversion to the desired product and the reaction mixture was purified directly using Accqprep (HpH Method D) and the relevant fractions combined and concentrated *in vacuo* to give the title product as a colourless gum (23.3 mg, 19 µmol, 41% yield).

^{1}H NMR: (600 MHz, DMSO-d_6) δ 8.97 (s, 1H), 8.49 (t, *J* = 6.0 Hz, 1H), 8.02 (d, *J* = 2.2 Hz, 1H), 7.84 (t, *J* = 5.6 Hz, 1H), 7.68 (d, *J* = 8.4 Hz, 1H), 7.66 (dd, *J* = 2.5, 1.1 Hz, 1H), 7.43 (d, *J* = 1.1 Hz, 1H), 7.40 (d, *J* = 7.6 Hz, 1H), 7.28 (dd, *J* = 9.1, 2.5 Hz, 1H), 7.05 (dd, *J* = 8.4, 1.5 Hz, 1H), 6.99 (d, *J* = 1.5 Hz, 1H), 6.94 (dd, *J* = 7.6, 1.5 Hz, 1H), 5.18 (d, *J* = 3.3 Hz, 1H), 4.81 (tt, *J* = 8.8, 4.6 Hz, 1H), 4.60 (d, *J* = 9.1 Hz, 1H), 4.52 (t, *J* = 8.2 Hz, 1H), 4.35 (br s, 1H), 4.29 (dd, *J* = 16.3, 6.2 Hz, 1H), 4.19 (dd, *J* = 16.7, 5.8 Hz, 1H), 4.05 – 3.96 (m, 4H), 3.75 (dd, *J* = 10.4, 4.5 Hz, 2H), 3.67 – 3.63 (m, 1H), 3.62 – 3.58 (m, 1H), 3.53 (s, 3H), 3.32 – 3.27 (m, 2H), 3.15 (s, 6H), 2.79 (t, *J* = 7.3 Hz, 2H), 2.46 – 2.44 (m, 3H), 2.08 (s, 3H), 2.02 (t, *J* = 7.4 Hz, 2H), 1.99 – 1.86 (m, 1H), 1.77 – 1.70 (m, 2H), 1.48 – 1.40 (m, 4H), 1.39 – 1.27 (m, 5H), 1.27 – 1.17 (m, 20H), 0.98 – 0.93 (m, 9H); **^{13}C{^{1}H} NMR:** (151 MHz, DMSO-d_6) δ 172.0, 171.8, 168.9, 168.0 (d, *J* = 20.7 Hz, 1C), 161.7, 155.8, 151.7, 151.4, 147.8, 143.3, 139.0, 137.3, 133.2, 132.1, 131.3, 130.8, 127.6, 127.4, 126.9, 123.1, 120.6, 118.8, 112.1, 111.6, 108.0, 78.9, 77.3, 69.7, 68.3 (d, *J* = 185.8 Hz, 1C), 58.8, 58.4, 56.8, 56.7, 56.5, 40.6, 37.9, 37.5, 37.2, 36.0, 35.4, 35.3, 29.1, 29.0, 29.0, 28.9, 28.9 (dd, *J* = 39.0, 4.4 Hz, 2C), 28.6, 28.6, 26.2, 26.1, 25.6, 25.3, 17.0, 16.0, 16.0, 12.8 (dd, *J* = 39.0, 10.4 Hz, 2C); **^{19}F{^{1}H} NMR:** (376 MHz, DMSO-d_6) δ -196.23 (s, 1F); **LCMS:** t_R = 1.50 min (100% purity by TAC) using 2 min HpH method as described in general experimental, $[M+H]^+$ 1153.6; **HRMS (ESI-TOF) m/z:** $[M+H]^+$ Calcd for $C_{63}H_{90}FN_8O_9S$ 1153.6530; found 1153.6516.

(2*S*,4*R*)-*N*-(2-((1-(1-(1,3-Dimethoxypropan-2-yl)-2-(1,5-dimethyl-6-oxo-1,6-dihydropyridin-3-yl)-1*H*-benzo[*d*]imidazol-5-yl)-4-oxo-6,9,12,15,18-pentaoxa-3-azaicosan-20-yl)oxy)-4-(4-methylthiazol-5-yl)benzyl)-1-((*S*)-2-(1-fluorocyclopropane-1-carboxamido)-3,3-dimethylbutanoyl)-4-hydroxypyrrolidine-2-carboxamide

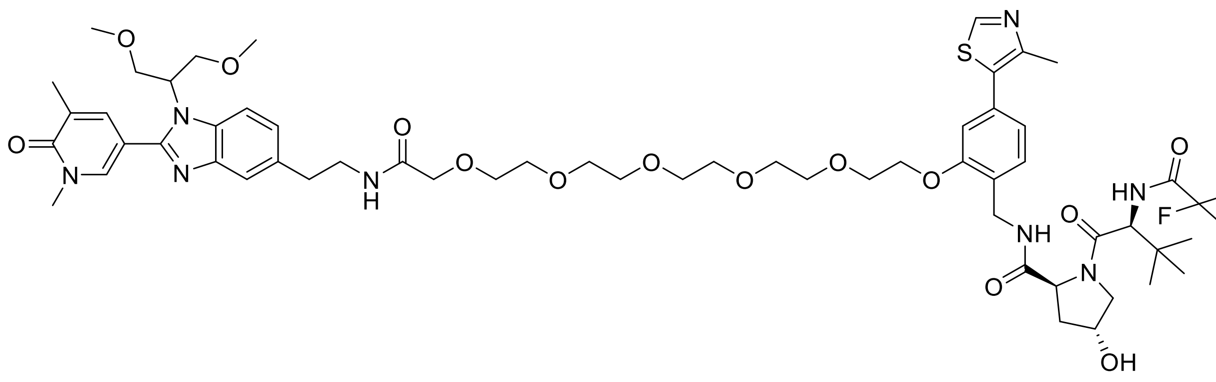

3e

To a solution of 17-(2-(((2*S*,4*R*)-1-((*S*)-2-(1-fluorocyclopropane-1-carboxamido)-3,3-dimethylbutanoyl)-4-hydroxypyrrolidine-2-carboxamido)methyl)-5-(4-methylthiazol-5-yl)phenoxy)-3,6,9,12,15-pentaoxaheptadecanoic acid (56.5 mg, 464.2 µL, 0.150 molar, 1.5 Eq, 69.629 µmol) in NMP was added a solution of 5-(5-(2-aminoethyl)-1-(1,3-dimethoxypropan-2-yl)-1*H*-benzo[*d*]imidazol-2-yl)-1,3-dimethylpyridin-2(1*H*)-one, hydrochloride (19.5 mg, 464.2 µL, 0.100 molar, 1 Eq, 46.419 µmol) in NMP, EDC (13.3 mg, 395.6 µL, 0.176 molar, 1.5 Eq, 69.629 µmol) in NMP, cyanic (*E*)-2-(hydroxyimino)butanoic anhydride (13.2 mg, 182.4 µL, 0.509 molar, 2 Eq, 92.838 µmol) in NMP and *N*-methylmorpholine (37.6 mg, 40.8 µL, 8 Eq, 371.35 µmol) and the reaction mixture was stirred at room temperature for 18 h in a sealed microwave vial. The reaction mixture was purified directly using Accqprep (HpH Method C) and the relevant fractions combined and concentrated *in vacuo* to give the title product as a colourless gum (23.3 mg, 19 µmol, 41% yield).

^{1}H NMR: (600 MHz, DMSO-d_6) δ 8.97 (s, 1H), 8.49 (t, *J* = 6.0 Hz, 1H), 8.02 (d, *J* = 2.5 Hz, 1H), 7.72 (t, *J* = 5.8 Hz, 1H), 7.69 (d, *J* = 8.4 Hz, 1H), 7.66 (dd, *J* = 2.5, 1.1 Hz, 1H), 7.46 – 7.43 (m, 1H), 7.41 (d, *J* = 8.0 Hz, 1H), 7.29 (dd, *J* = 9.3, 2.7 Hz, 1H), 7.06 (dd, *J* = 8.5, 1.6 Hz, 1H), 7.03 (d, *J* = 1.8 Hz, 1H), 6.96 (dd, *J* = 7.8, 1.6 Hz, 1H), 5.17 (d, *J* = 3.6 Hz, 1H), 4.81 (dt, *J* = 8.7, 4.4 Hz, 1H), 4.60 (d, *J* = 9.1 Hz, 1H), 4.52 (t, *J* = 8.2 Hz, 1H), 4.35 (br s, 1H), 4.33 – 4.28 (m, 1H), 4.23 – 4.16 (m, 3H), 3.99 (dd, *J* = 10.2, 9.1 Hz, 2H), 3.85 (s, 2H), 3.79 – 3.73 (m, 4H), 3.67 – 3.63 (m, 1H), 3.62 – 3.59 (m, 3H), 3.55 – 3.46 (m, 18H), 3.41 – 3.36 (m, 2H), 3.15 (s, 6H), 2.84 (t, *J* = 7.4 Hz, 2H), 2.47 – 2.44 (m, 3H), 2.08 (s, 3H), 1.92 (ddd, *J* = 13.1, 8.7, 4.4 Hz, 1H), 1.41 – 1.30 (m, 2H), 1.25 – 1.20 (m, 2H), 0.96 (s, 9H); **^{13}C{^{1}H} NMR:** (151 MHz, DMSO-d_6) δ 172.3, 169.6, 169.4, 168.5 (d, *J* = 20.2 Hz, 1C), 162.2, 156.3, 152.3, 151.9, 151.9, 148.4, 143.8, 139.5, 137.8, 133.5, 132.6, 131.8, 131.4, 128.2, 128.0, 127.6, 123.6, 121.5, 119.3, 112.7, 112.6, 108.5, 79.4, 77.8, 70.7, 70.6, 70.4, 70.3, 70.3, 70.3, 70.2, 70.2, 70.0, 69.4, 68.9 (d, *J* = 166.8 Hz, 1C), 59.3, 58.9, 57.3, 57.2, 57.0, 40.7, 38.4, 38.0, 37.7, 36.6, 35.7, 26.7, 17.5, 16.5, 13.3 (dd, *J* = 39.8, 9.8 Hz, 2C); **^{19}F{^{1}H} NMR:** (376 MHz, DMSO-d_6) δ -196.21 (s, 1F); **LCMS:** t_R = 1.00 min (98% purity by TAC) using 2 min HpH

method as described in general experimental, [M+H]$^+$ 1177.4; **HRMS (ESI-TOF) m/z:** [M+H]$^+$ Calcd for $C_{59}H_{82}FN_8O_{14}S$ 1177.5650; found 1177.5621.

NMR Spectra

Batch Scale Resynthesis

Compound 3a – ^{1}H NMR (600 MHz, DMSO-d_6)

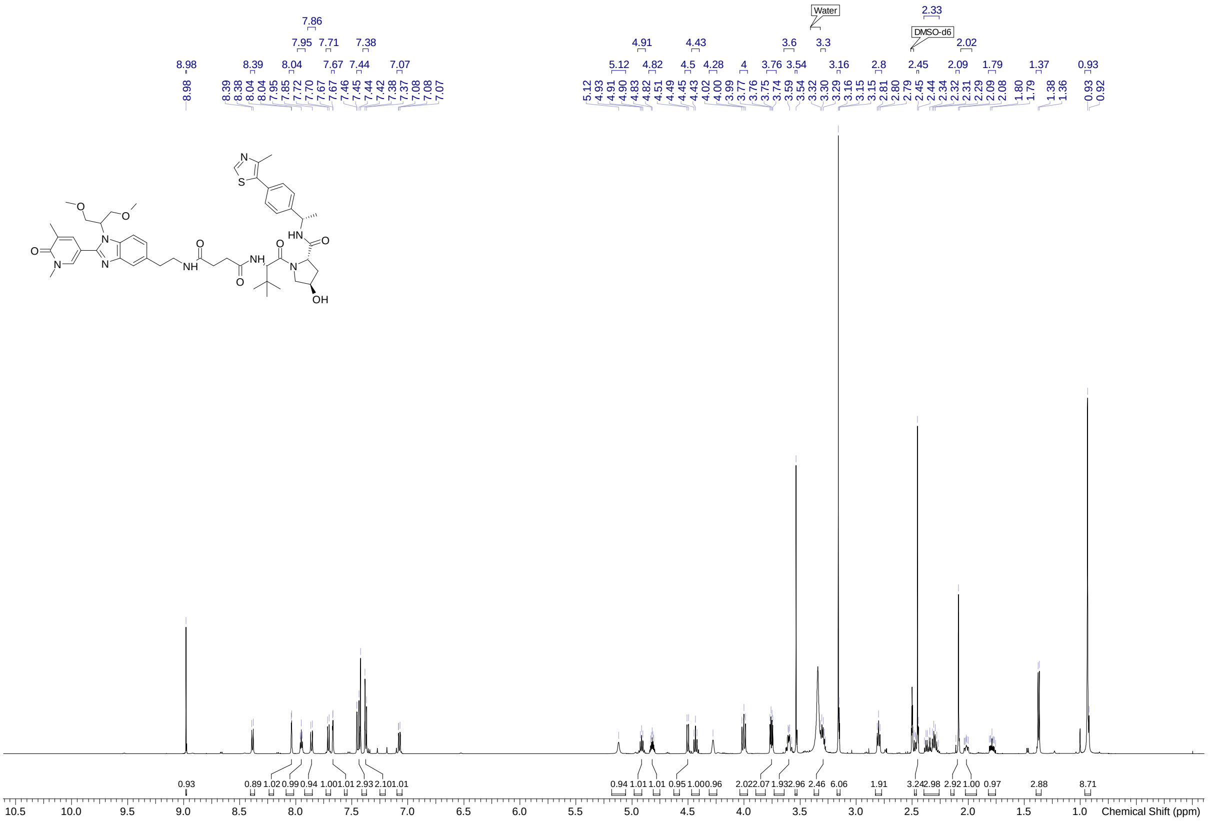

Compound 3a – $^{13}C\{^{1}H\}$ NMR (151 MHz, DMSO-d_6)

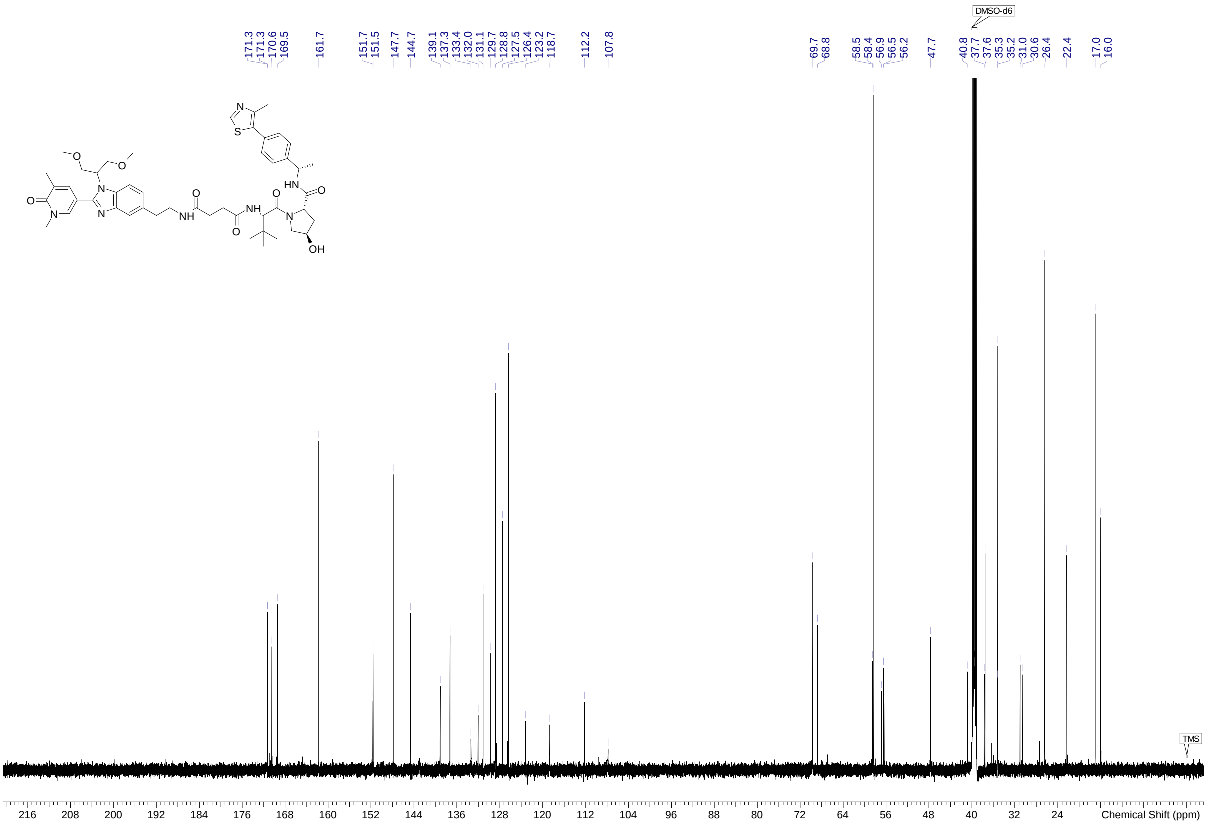

Compound 3b – ^{1}H NMR (600 MHz, DMSO-d_6)

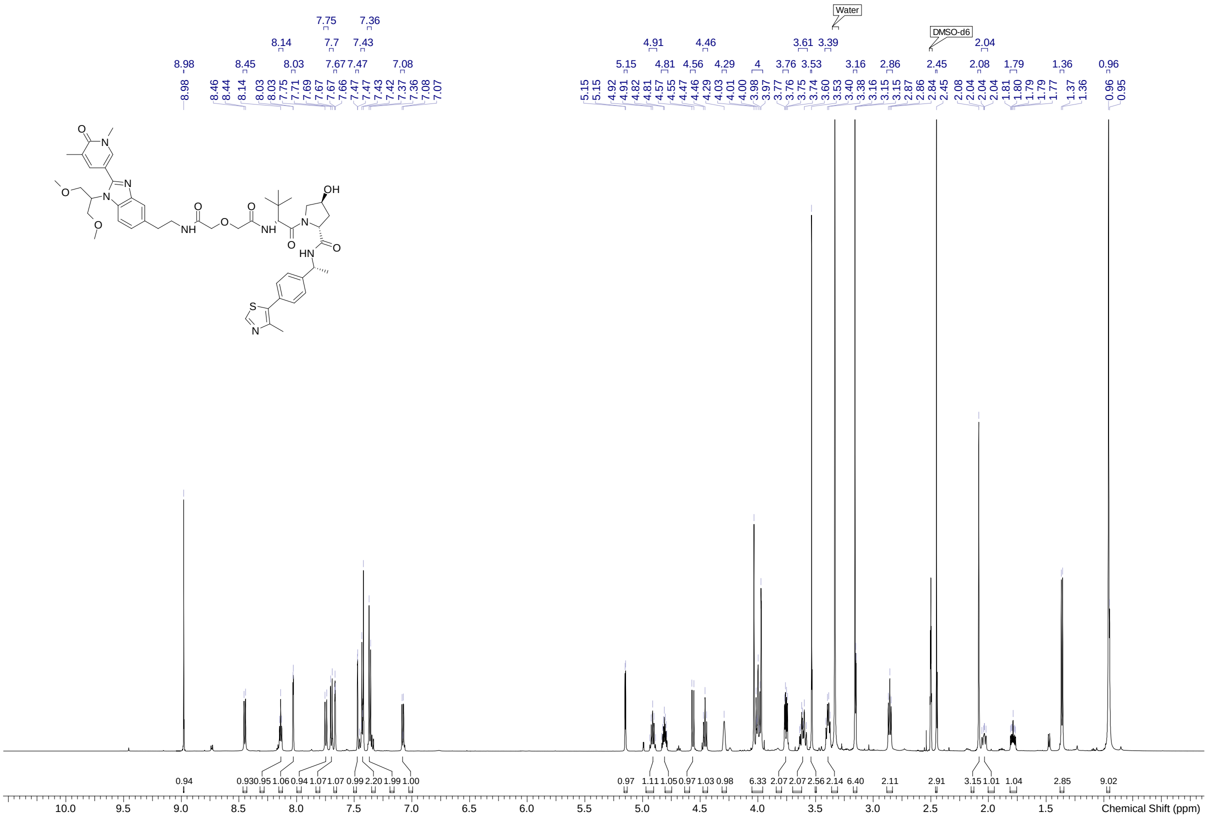

Compound 3b – ^{13}C {^{1}H} NMR (151 MHz, DMSO-d_6)

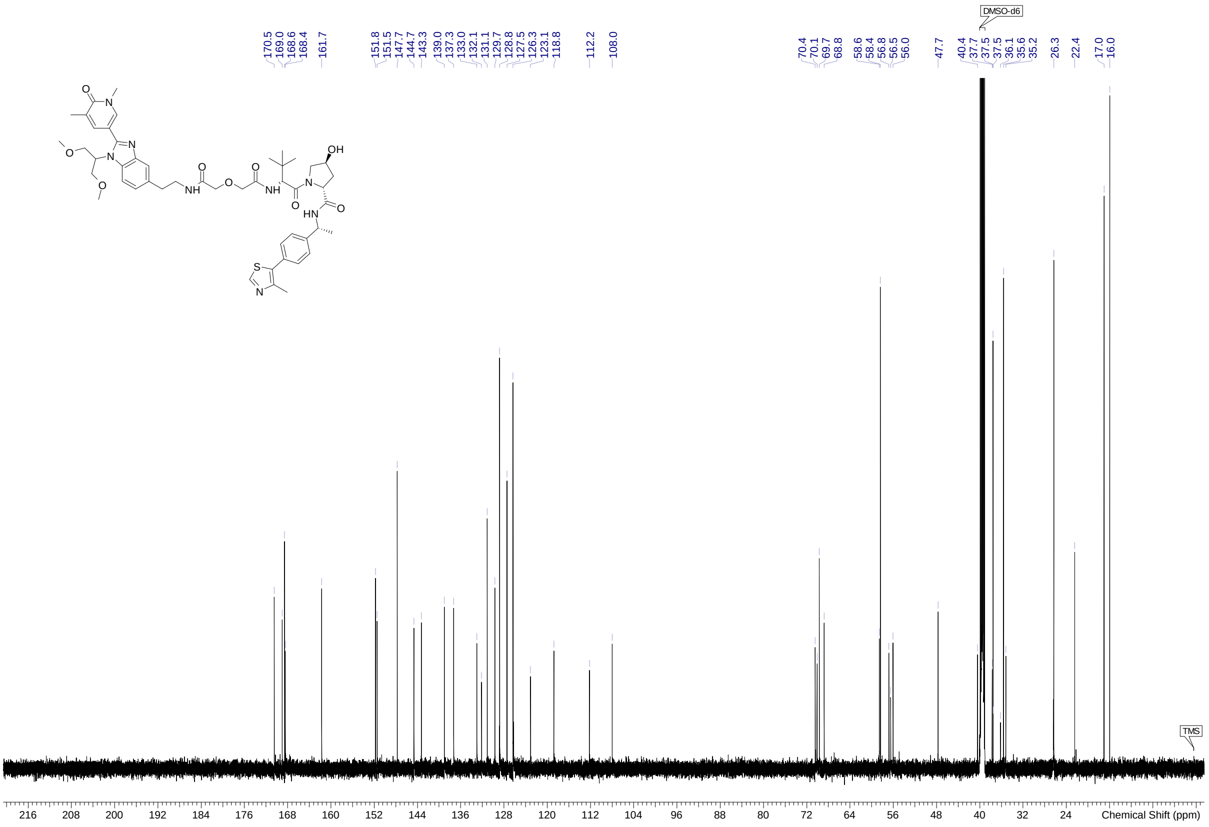

Compound 3c – ^{1}H NMR (600 MHz, DMSO-d_6)

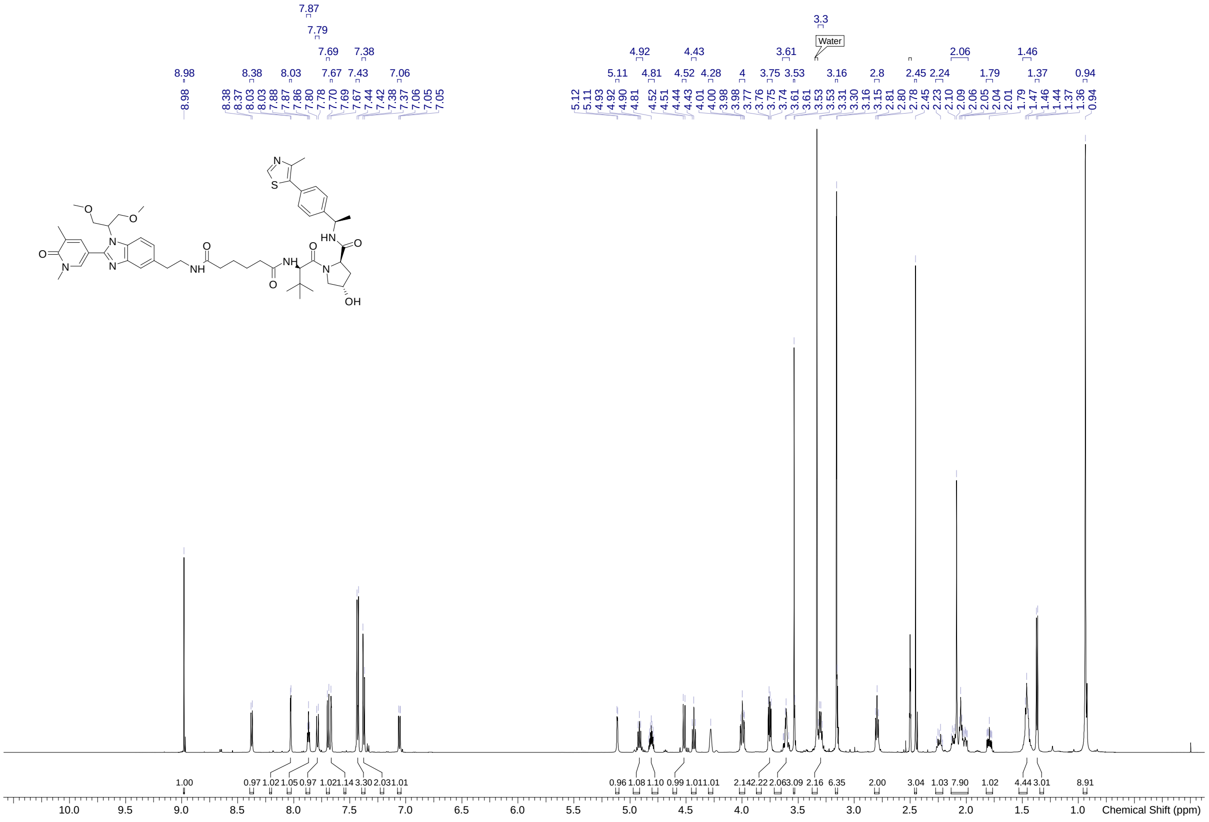

Compound 3c – ^{13}C {^{1}H} NMR (151 MHz, DMSO-d_6)

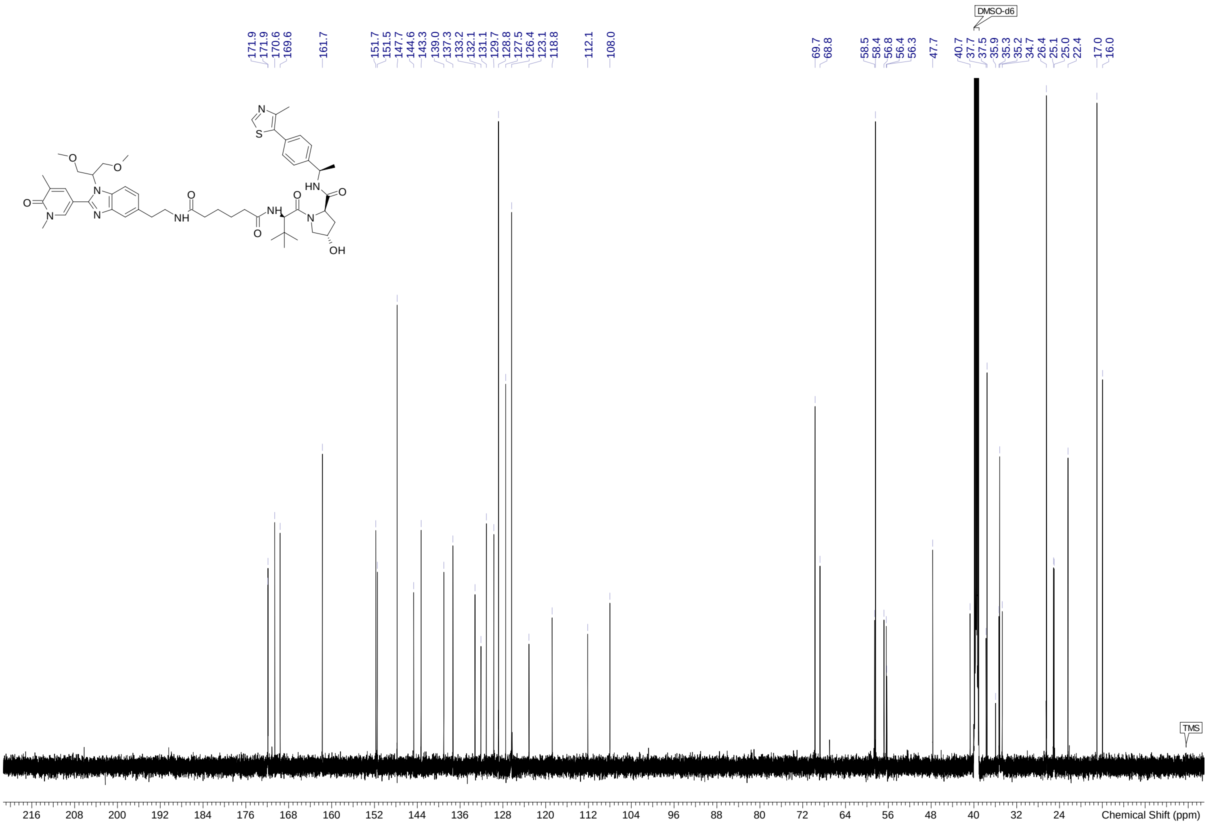

Compound 3d – ^{1}H NMR (600 MHz, DMSO-d_6)

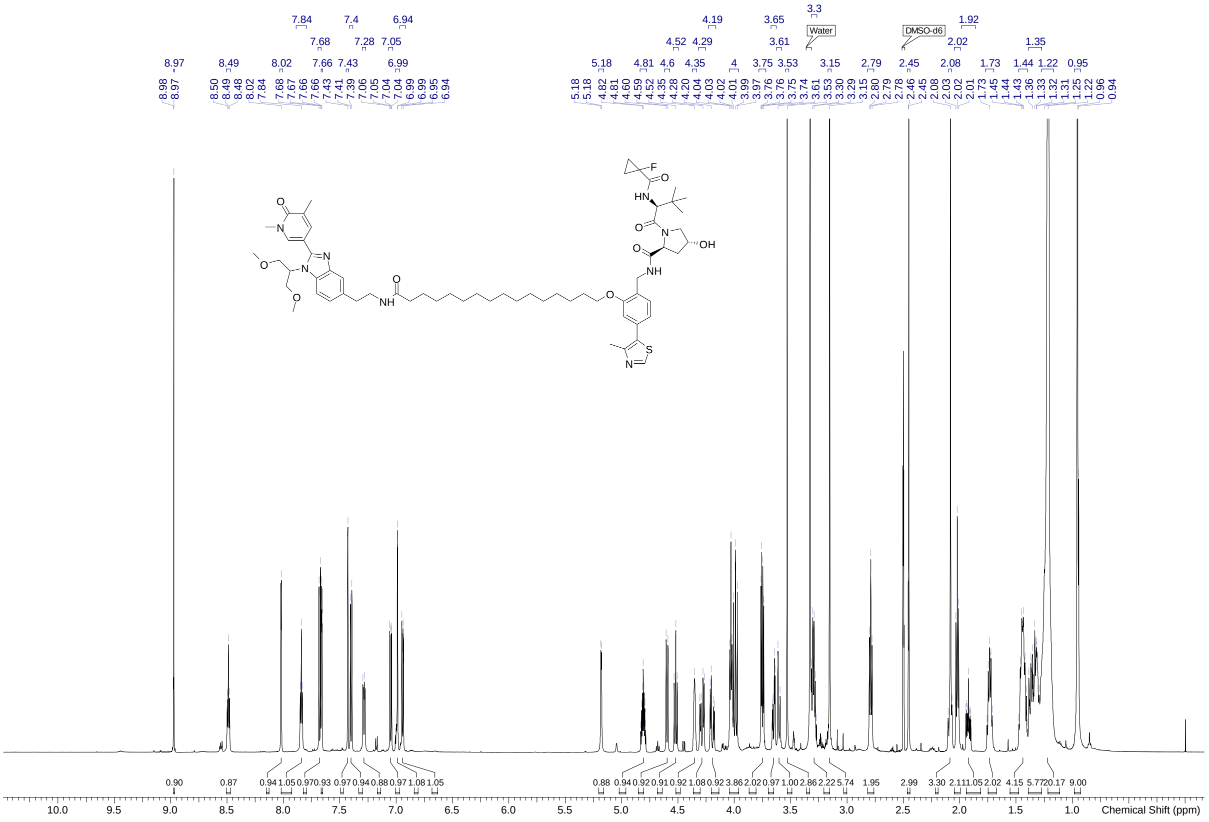

Compound 3d – ^{13}C {^{1}H} NMR (151 MHz, DMSO-d_6)

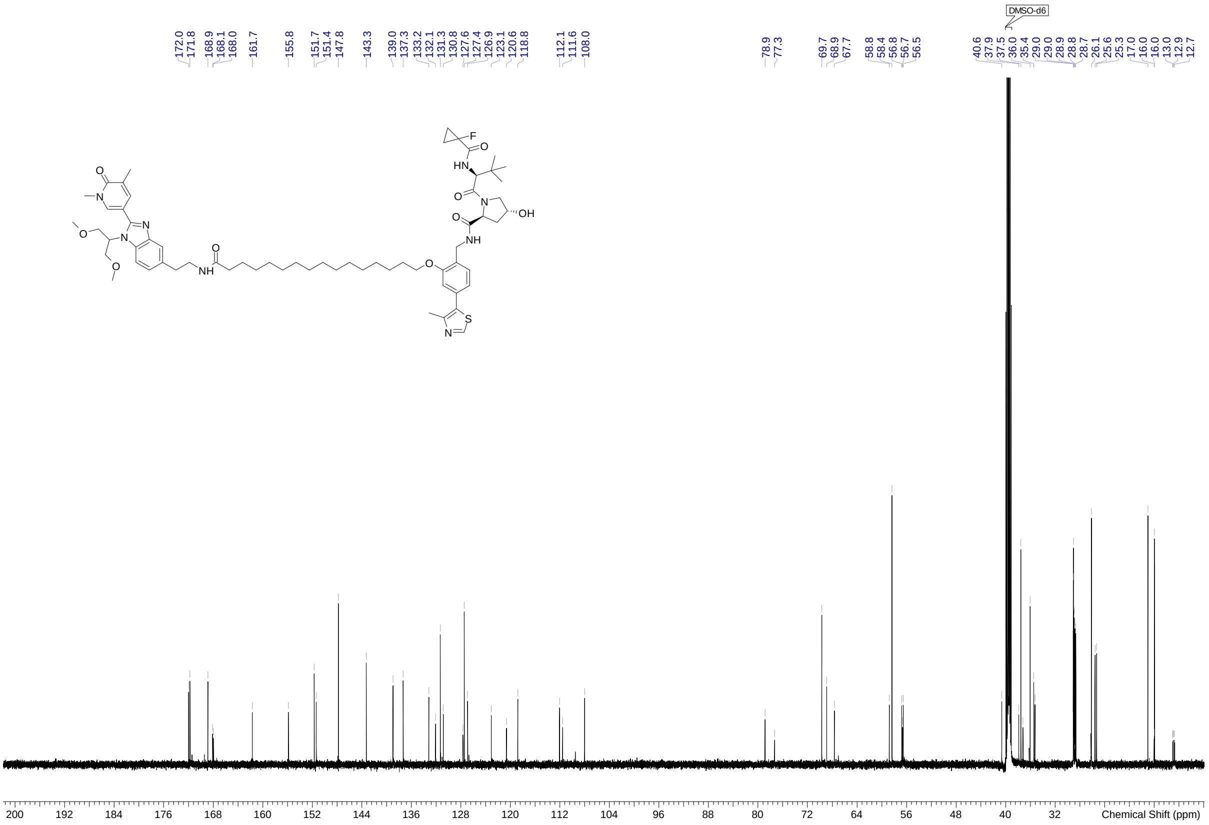

Compound 3d – ^{19}F NMR (376 MHz, DMSO-d_6)

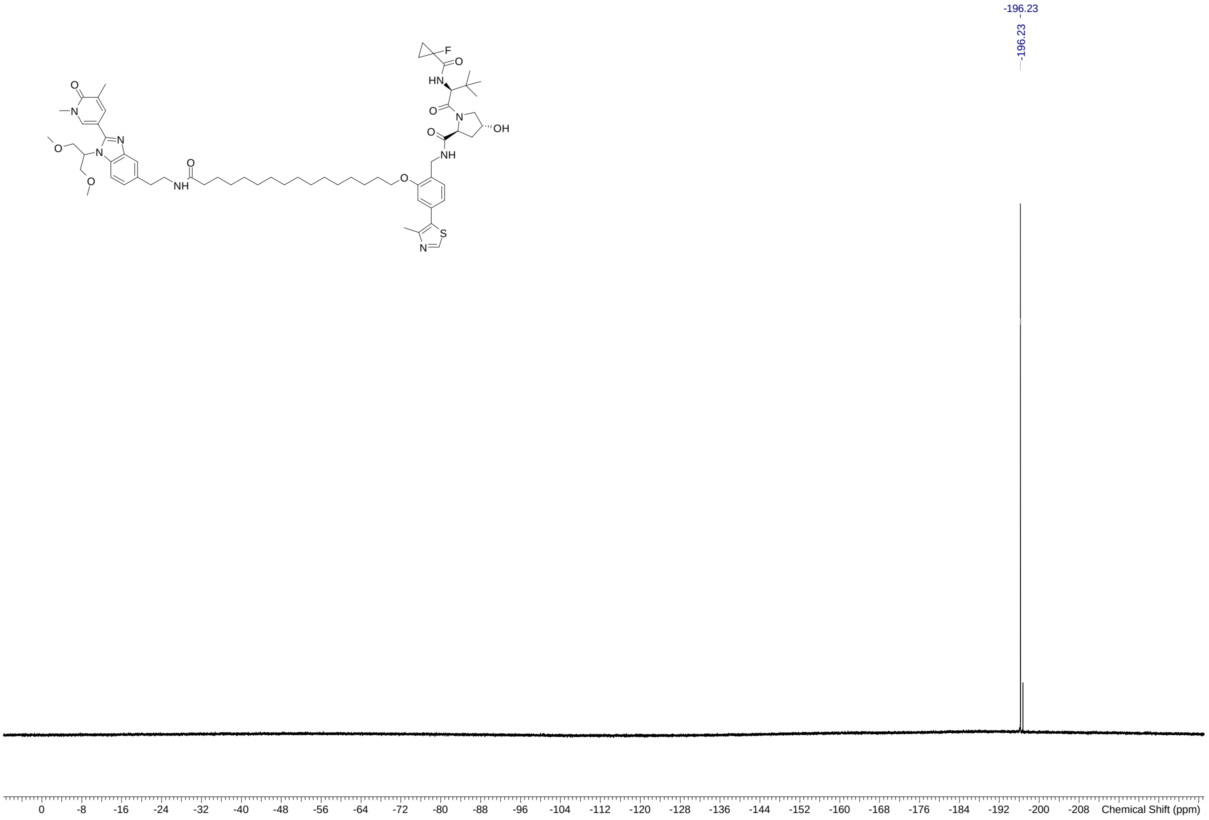

Compound 3e – ^{1}H NMR (600 MHz, DMSO-d_6)

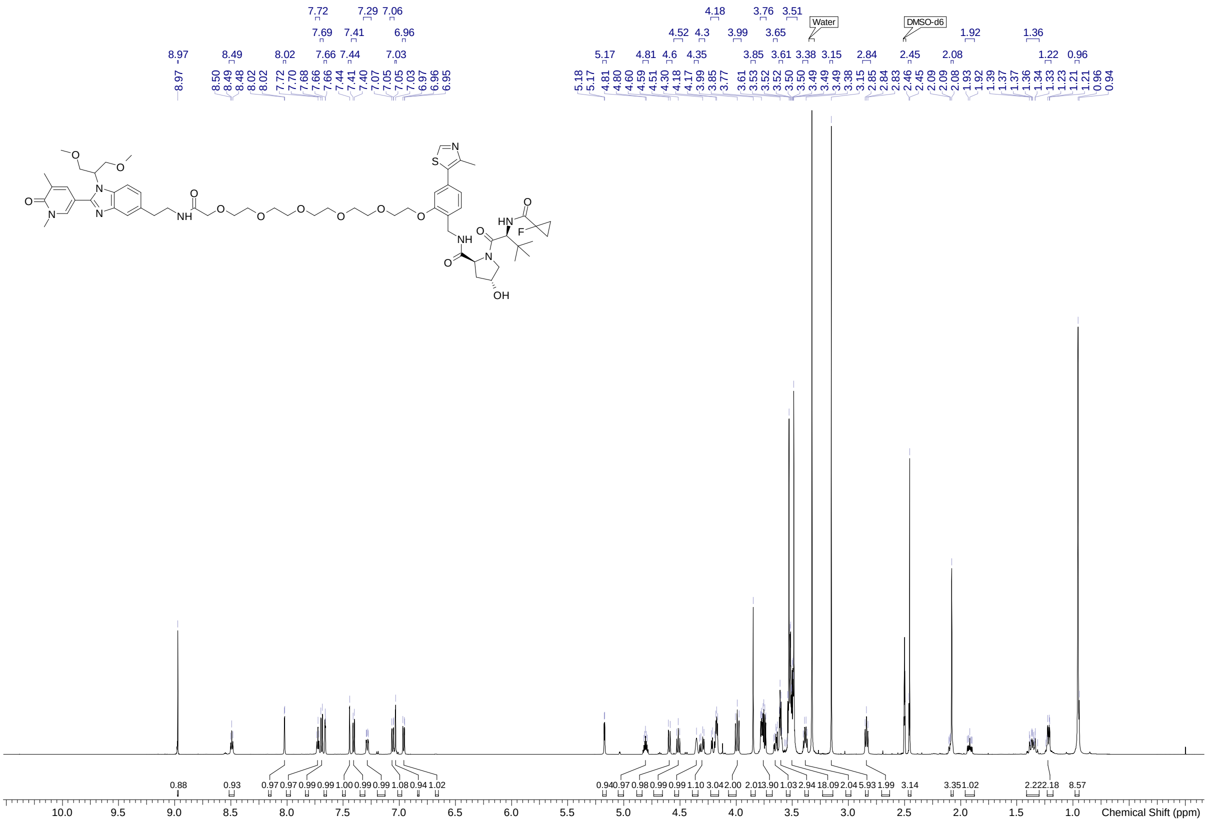

Compound 3e – ^{13}C {^{1}H} NMR (151 MHz, DMSO-d_6)

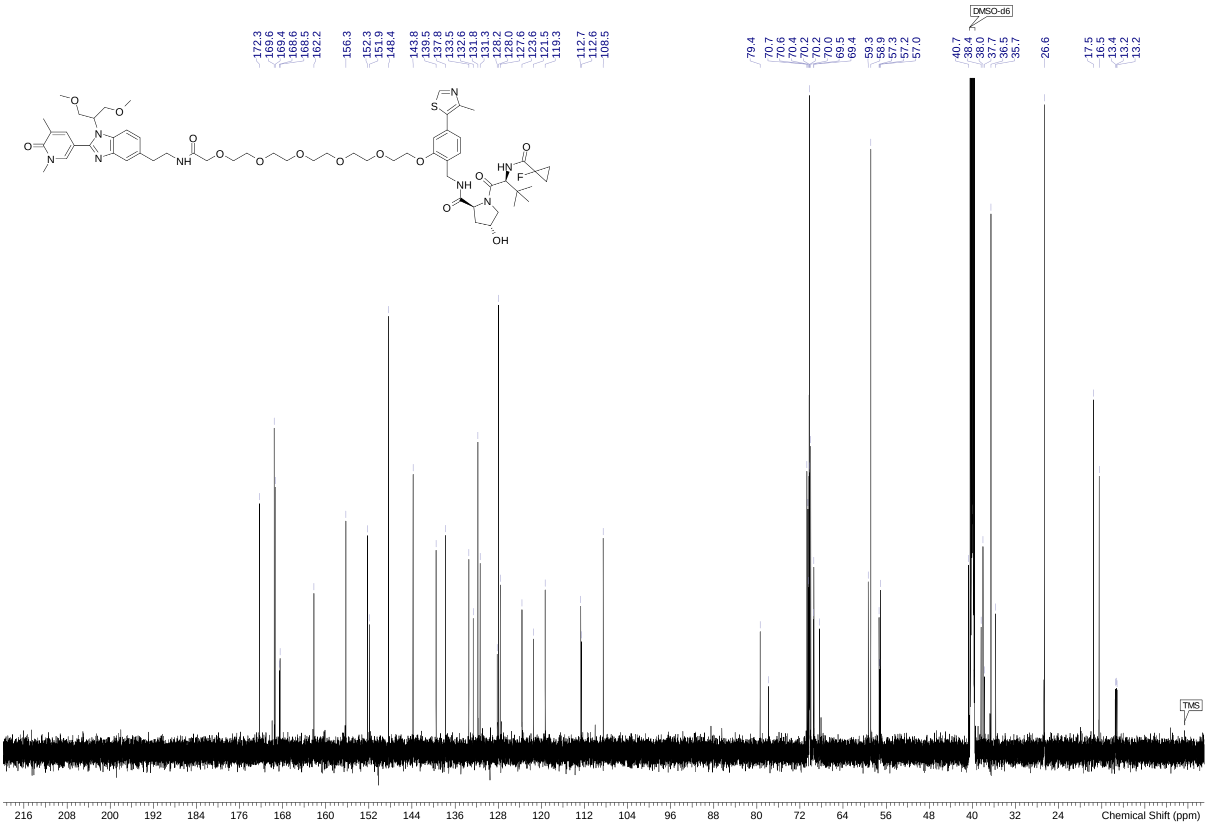

Compound 3e – ^{19}F NMR (376 MHz, DMSO-d_6)

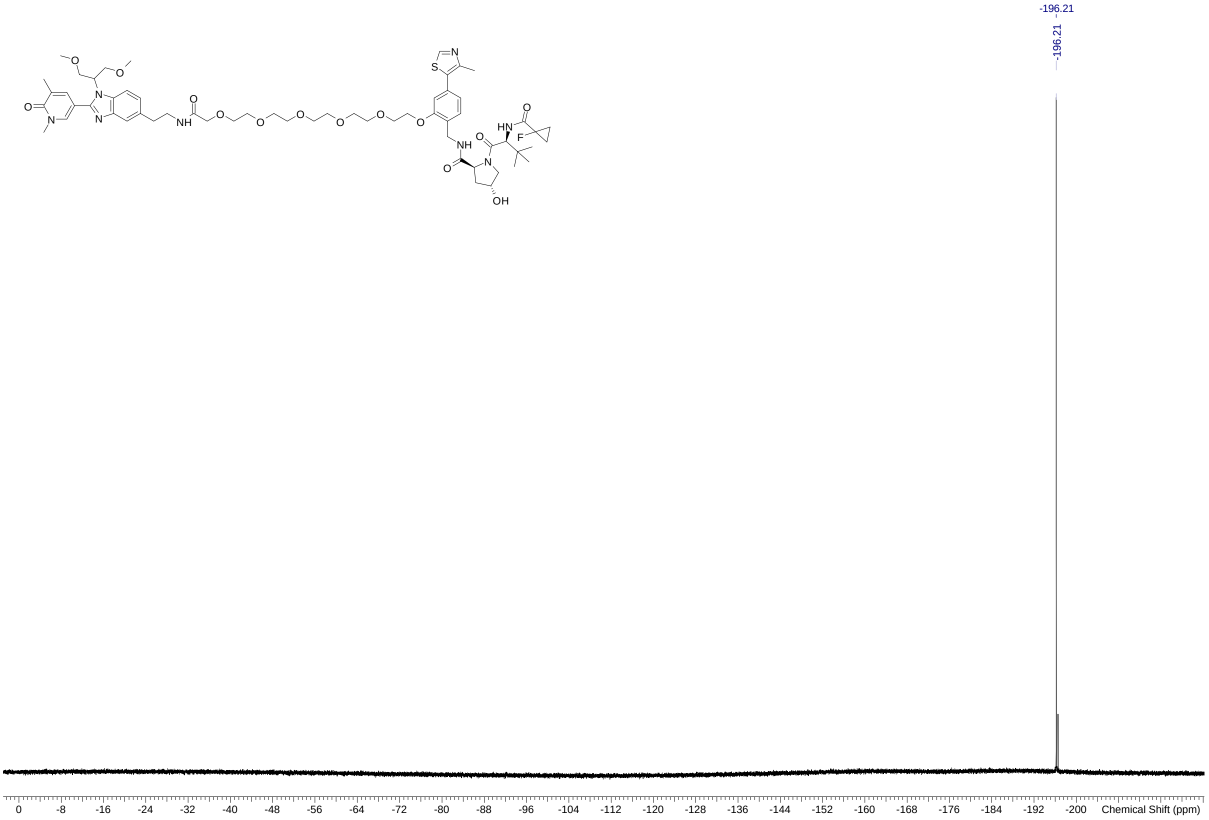

Data Tables

Figure 2A

Library monomer	Product peak area / % TFA salt	HCl salt	Free amine
1	59	60	58
2	2	6	40
3	9	18	43
4	12	16	22
5	7	14	44
6	40	32	63
7	9	19	43
8	4	5	17
9	9	15	39
10	11	17	41
11	9	16	43
12	12	16	50
13	9	16	44
14	42	33	66
15	4	4	7
16	29	16	21
17	42	36	61
18	8	15	47
19	11	18	53
20	12	15	48
21	14	17	52
22	10	19	46
23	10	17	48
24	19	18	48
25	15	19	50
26	15	13	9
27	7	15	21
28	9	7	30
29	16	20	48
30	10	17	50
31	5	6	24
32	38	65	73
33	44	12	31
34	14	3	23
35	10	8	43
36	20	14	54
37	15	13	47
38	17	10	49
39	17	11	48
40	20	17	47
41	21	14	62
42	12	4	26
43	10	13	55
44	41	19	48
45	2	0	7
46	16	6	29
47	12	4	23
48	29	5	24
49	13	12	48
50	21	16	14
51	24	10	71
52	19	16	50
53	19	16	48
54	10	9	37
55	16	7	27
56	0	3	10
57	22	16	48
58	21	14	50
59	15	14	38
60	19	9	25

61	13	12	50
62	25	32	59
63	16	14	47
64	16	11	27
65	3	9	26
66	8	13	36
67	9	16	40
68	9	18	42
69	9	19	42
70	10	14	40
71	9	18	41
72	10	16	34
73	9	14	38
74	7	17	39
75	8	14	42
76	8	15	42
77	9	15	41
78	7	3	38
79	8	14	33
80	9	17	42
81	12	15	48
82	15	17	50
83	15	17	44
84	12	16	50
85	7	10	36
86	17	18	50
87	12	16	49

Table S1. Data to support Figure 2A. Product peak area values were calculated by PyParse, an automated LCMS analysis software.

Figure 2B

Product purity by LCMS / %	Free amine	TFA salt
0	36	517
1 - 25	113	128
26 - 50	107	44
51-75	418	7
76 - 100	22	0

Table S2. Processed data to support Figure 2B; unprocessed data shown in Table S3.

Amine	Library monomer	Product peak area with free amine / %	Product peak area with TFA salt / %
**1a**	1	62	0
**1a**	2	54	0
**1a**	3	63	0
**1a**	4	60	0
**1a**	5	84	0
**1a**	6	60	0
**1a**	7	79	0
**1a**	8	12	0
**1a**	9	59	0
**1a**	10	75	0
**1a**	11	61	0
**1a**	12	64	0
**1a**	13	55	0
**1a**	14	3	0
**1a**	15	0	0
**1a**	16	58	0
**1a**	17	3	0
**1a**	18	59	0
**1a**	19	69	0
**1a**	20	61	0
**1a**	21	62	0
**1a**	22	2	51
**1a**	23	65	0
**1a**	24	2	0
**1a**	25	48	0

**1a**	26	32	0
**1a**	27	59	0
**1a**	28	57	0
**1a**	29	69	0
**1a**	30	2	0
**1a**	31	4	0
**1a**	32	60	0
**1a**	33	47	0
**1a**	34	60	0
**1a**	35	78	0
**1a**	36	60	0
**1a**	37	57	0
**1a**	38	62	0
**1a**	39	59	0
**1a**	40	59	0
**1a**	41	54	0
**1a**	42	64	41
**1a**	43	63	0
**1a**	44	57	18
**1a**	45	8	0
**1a**	46	8	0
**1a**	47	19	0
**1a**	48	31	0
**1a**	49	65	0
**1a**	50	63	0
**1a**	51	74	0
**1a**	52	61	0
**1a**	53	62	0
**1a**	54	0	0
**1a**	55	29	0
**1a**	56	0	0
**1a**	57	2	0
**1a**	58	2	0

**1a**	59	7	0
**1a**	60	0	0
**1a**	61	67	0
**1a**	62	14	0
**1a**	63	76	0
**1a**	64	2	0
**1a**	65	39	0
**1a**	66	72	0
**1a**	67	72	0
**1a**	68	66	0
**1a**	69	2	0
**1a**	70	63	0
**1a**	71	62	0
**1a**	72	42	0
**1a**	73	60	0
**1a**	74	3	0
**1a**	75	65	0
**1a**	76	60	0
**1a**	77	57	0
**1a**	78	35	0
**1a**	79	51	0
**1a**	80	60	0
**1a**	81	62	0
**1a**	82	2	0
**1a**	83	30	1
**1a**	84	65	0
**1a**	85	6	0
**1a**	86	60	0
**1a**	87	30	36
**1b**	1	76	0
**1b**	2	57	38
**1b**	3	70	0
**1b**	4	67	0

**1b**	5	67	0
**1b**	6	83	0
**1b**	7	66	0
**1b**	8	34	0
**1b**	9	62	0
**1b**	10	57	0
**1b**	11	18	0
**1b**	12	70	0
**1b**	13	64	0
**1b**	14	85	0
**1b**	15	0	0
**1b**	16	74	0
**1b**	17	88	0
**1b**	18	63	0
**1b**	19	62	0
**1b**	20	59	0
**1b**	21	68	0
**1b**	22	66	0
**1b**	23	69	0
**1b**	24	60	0
**1b**	25	55	0
**1b**	26	31	0
**1b**	27	69	0
**1b**	28	66	0
**1b**	29	67	0
**1b**	30	65	0
**1b**	31	21	0
**1b**	32	100	0
**1b**	33	55	26
**1b**	34	63	0
**1b**	35	55	0
**1b**	36	67	0
**1b**	37	53	0
**1b**	38	69	0
**1b**	39	64	0
**1b**	40	59	0
**1b**	41	58	0
**1b**	42	10	0
**1b**	43	58	0
**1b**	44	83	44
**1b**	45	12	0
**1b**	46	14	0
**1b**	47	19	0
**1b**	48	29	0
**1b**	49	50	0
**1b**	50	6	0
**1b**	51	68	0
**1b**	52	69	0
**1b**	53	67	0
**1b**	54	2	0
**1b**	55	36	0
**1b**	56	0	0
**1b**	57	64	0
**1b**	58	72	0
**1b**	59	13	0
**1b**	60	0	0
**1b**	61	62	0
**1b**	62	26	0
**1b**	63	65	0
**1b**	64	3	0
**1b**	65	45	0
**1b**	66	59	0
**1b**	67	65	0
**1b**	68	69	0
**1b**	69	67	0
**1b**	70	67	0

**1b**	71	69	39
**1b**	72	56	0
**1b**	73	68	0
**1b**	74	62	0
**1b**	75	66	0
**1b**	76	66	0
**1b**	77	63	0
**1b**	78	43	0
**1b**	79	60	0
**1b**	80	69	0
**1b**	81	59	0
**1b**	82	65	52
**1b**	83	34	0
**1b**	84	71	0
**1b**	85	11	0
**1b**	86	68	7
**1b**	87	39	0
**1c**	1	0	0
**1c**	2	49	0
**1c**	3	63	0
**1c**	4	77	0
**1c**	5	0	0
**1c**	6	63	0
**1c**	7	58	0
**1c**	8	12	0
**1c**	9	54	35
**1c**	10	54	31
**1c**	11	19	39
**1c**	12	60	0
**1c**	13	57	0
**1c**	14	61	0
**1c**	15	0	0
**1c**	16	58	0
**1c**	17	80	0
**1c**	18	20	0
**1c**	19	52	0
**1c**	20	48	0
**1c**	21	56	0
**1c**	22	56	2
**1c**	23	59	0
**1c**	24	49	0
**1c**	25	43	0
**1c**	26	23	0
**1c**	27	66	0
**1c**	28	54	0
**1c**	29	65	0
**1c**	30	55	0
**1c**	31	6	0
**1c**	32	63	0
**1c**	33	45	28
**1c**	34	55	0
**1c**	35	53	0
**1c**	36	49	0
**1c**	37	54	0
**1c**	38	60	0
**1c**	39	55	0
**1c**	40	56	0
**1c**	41	58	0
**1c**	42	67	0
**1c**	43	58	0
**1c**	44	17	20
**1c**	45	27	0
**1c**	46	7	0
**1c**	47	22	0
**1c**	48	25	0
**1c**	49	59	0

**1c**	50	3	0
**1c**	51	83	0
**1c**	52	59	0
**1c**	53	60	0
**1c**	54	0	0
**1c**	55	30	0
**1c**	56	0	0
**1c**	57	72	0
**1c**	58	65	0
**1c**	59	5	0
**1c**	60	0	0
**1c**	61	65	0
**1c**	62	38	0
**1c**	63	57	0
**1c**	64	0	0
**1c**	65	35	0
**1c**	66	50	0
**1c**	67	55	0
**1c**	68	64	0
**1c**	69	59	0
**1c**	70	59	0
**1c**	71	62	0
**1c**	72	26	0
**1c**	73	58	0
**1c**	74	53	0
**1c**	75	58	0
**1c**	76	60	0
**1c**	77	55	0
**1c**	78	38	0
**1c**	79	50	0
**1c**	80	60	0
**1c**	81	51	0
**1c**	82	58	0

**1c**	83	33	51
**1c**	84	62	0
**1c**	85	7	0
**1c**	86	58	0
**1c**	87	33	0
**1d**	1	56	10
**1d**	2	2	0
**1d**	3	69	0
**1d**	4	58	0
**1d**	5	61	33
**1d**	6	57	0
**1d**	7	56	0
**1d**	8	56	0
**1d**	9	17	17
**1d**	10	61	15
**1d**	11	56	0
**1d**	12	69	42
**1d**	13	68	35
**1d**	14	58	17
**1d**	15	0	0
**1d**	16	61	0
**1d**	17	59	21
**1d**	18	54	46
**1d**	19	12	7
**1d**	20	15	15
**1d**	21	58	53
**1d**	22	19	0
**1d**	23	60	17
**1d**	24	14	12
**1d**	25	50	6
**1d**	26	39	0
**1d**	27	47	0
**1d**	28	57	0

**1d**	29	72	20
**1d**	30	14	0
**1d**	31	12	39
**1d**	32	56	0
**1d**	33	43	20
**1d**	34	48	0
**1d**	35	49	0
**1d**	36	58	0
**1d**	37	55	10
**1d**	38	61	19
**1d**	39	55	0
**1d**	40	55	7
**1d**	41	65	17
**1d**	42	65	0
**1d**	43	57	7
**1d**	44	51	13
**1d**	45	7	0
**1d**	46	13	47
**1d**	47	34	0
**1d**	48	49	26
**1d**	49	61	0
**1d**	50	2	2
**1d**	51	62	3
**1d**	52	59	0
**1d**	53	59	0
**1d**	54	7	0
**1d**	55	35	0
**1d**	56	0	0
**1d**	57	78	0
**1d**	58	3	0
**1d**	59	6	0
**1d**	60	4	0
**1d**	61	78	2

**1d**	62	9	0
**1d**	63	54	38
**1d**	64	8	0
**1d**	65	55	0
**1d**	66	52	0
**1d**	67	56	0
**1d**	68	82	3
**1d**	69	59	3
**1d**	70	49	3
**1d**	71	63	0
**1d**	72	44	0
**1d**	73	61	0
**1d**	74	48	2
**1d**	75	59	2
**1d**	76	56	0
**1d**	77	55	0
**1d**	78	40	0
**1d**	79	55	0
**1d**	80	60	1
**1d**	81	54	3
**1d**	82	59	0
**1d**	83	33	2
**1d**	84	64	3
**1d**	85	7	0
**1d**	86	59	0
**1d**	87	38	2
**1e**	1	61	0
**1e**	2	38	0
**1e**	3	63	0
**1e**	4	30	0
**1e**	5	51	0
**1e**	6	44	38
**1e**	7	62	0

**1e**	8	7	0
**1e**	9	59	0
**1e**	10	59	0
**1e**	11	60	0
**1e**	12	60	0
**1e**	13	46	34
**1e**	14	51	34
**1e**	15	0	0
**1e**	16	44	0
**1e**	17	45	0
**1e**	18	43	0
**1e**	19	53	0
**1e**	20	48	0
**1e**	21	61	0
**1e**	22	59	0
**1e**	23	61	0
**1e**	24	51	0
**1e**	25	33	0
**1e**	26	19	0
**1e**	27	4	0
**1e**	28	42	0
**1e**	29	59	0
**1e**	30	56	0
**1e**	31	0	0
**1e**	32	61	0
**1e**	33	53	23
**1e**	34	27	0
**1e**	35	50	0
**1e**	36	55	0
**1e**	37	51	0
**1e**	38	63	0
**1e**	39	62	0
**1e**	40	58	0

**1e**	41	61	0
**1e**	42	68	0
**1e**	43	58	0
**1e**	44	74	23
**1e**	45	10	0
**1e**	46	2	0
**1e**	47	13	0
**1e**	48	35	0
**1e**	49	45	0
**1e**	50	63	0
**1e**	51	66	0
**1e**	52	63	0
**1e**	53	64	0
**1e**	54	3	0
**1e**	55	3	0
**1e**	56	0	0
**1e**	57	57	0
**1e**	58	66	0
**1e**	59	3	0
**1e**	60	6	0
**1e**	61	55	0
**1e**	62	8	0
**1e**	63	61	0
**1e**	64	5	0
**1e**	65	4	0
**1e**	66	56	0
**1e**	67	60	0
**1e**	68	55	0
**1e**	69	51	0
**1e**	70	55	0
**1e**	71	76	0
**1e**	72	0	0
**1e**	73	63	0

**1e**	74	47	0
**1e**	75	51	0
**1e**	76	64	0
**1e**	77	56	0
**1e**	78	32	0
**1e**	79	75	0
**1e**	80	63	0
**1e**	81	48	0
**1e**	82	62	0
**1e**	83	33	5
**1e**	84	53	0
**1e**	85	7	0
**1e**	86	61	1
**1e**	87	30	0
**1f**	1	66	13
**1f**	2	54	8
**1f**	3	75	13
**1f**	4	69	13
**1f**	5	67	8
**1f**	6	63	8
**1f**	7	67	13
**1f**	8	27	0
**1f**	9	65	48
**1f**	10	64	11
**1f**	11	66	15
**1f**	12	68	15
**1f**	13	67	28
**1f**	14	65	6
**1f**	15	0	0
**1f**	16	61	0
**1f**	17	66	8
**1f**	18	60	7
**1f**	19	59	65

**1f**	20	53	40
**1f**	21	2	53
**1f**	22	65	13
**1f**	23	71	9
**1f**	24	57	45
**1f**	25	53	6
**1f**	26	25	0
**1f**	27	73	0
**1f**	28	48	0
**1f**	29	73	49
**1f**	30	66	8
**1f**	31	38	0
**1f**	32	68	15
**1f**	33	55	20
**1f**	34	20	0
**1f**	35	62	25
**1f**	36	67	8
**1f**	37	60	31
**1f**	38	67	47
**1f**	39	65	12
**1f**	40	65	7
**1f**	41	4	0
**1f**	42	65	9
**1f**	43	64	5
**1f**	44	74	49
**1f**	45	9	0
**1f**	46	29	0
**1f**	47	28	0
**1f**	48	3	48
**1f**	49	64	9
**1f**	50	2	11
**1f**	51	72	16
**1f**	52	69	0

**1f**	53	68	16
**1f**	54	0	0
**1f**	55	48	0
**1f**	56	0	0
**1f**	57	63	12
**1f**	58	75	9
**1f**	59	22	0
**1f**	60	29	0
**1f**	61	68	9
**1f**	62	37	0
**1f**	63	66	0
**1f**	64	22	0
**1f**	65	41	17
**1f**	66	61	9
**1f**	67	65	13
**1f**	68	66	0
**1f**	69	64	10
**1f**	70	62	0
**1f**	71	77	50
**1f**	72	47	0
**1f**	73	77	0
**1f**	74	54	0
**1f**	75	68	10
**1f**	76	67	14
**1f**	77	65	16
**1f**	78	40	0
**1f**	79	72	43
**1f**	80	68	13
**1f**	81	65	0
**1f**	82	66	4
**1f**	83	31	4
**1f**	84	70	0
**1f**	85	9	0

**1f**	86	66	5
**1f**	87	34	0
**1g**	1	58	0
**1g**	2	45	0
**1g**	3	62	0
**1g**	4	57	0
**1g**	5	26	0
**1g**	6	52	0
**1g**	7	55	0
**1g**	8	14	0
**1g**	9	52	0
**1g**	10	76	0
**1g**	11	76	0
**1g**	12	59	0
**1g**	13	54	0
**1g**	14	63	0
**1g**	15	0	0
**1g**	16	56	0
**1g**	17	55	0
**1g**	18	49	0
**1g**	19	52	0
**1g**	20	52	0
**1g**	21	54	0
**1g**	22	62	0
**1g**	23	57	0
**1g**	24	52	0
**1g**	25	21	0
**1g**	26	20	0
**1g**	27	4	0
**1g**	28	50	0
**1g**	29	55	0
**1g**	30	75	0
**1g**	31	0	0

**1g**	32	56	0
**1g**	33	48	25
**1g**	34	10	0
**1g**	35	36	0
**1g**	36	48	0
**1g**	37	44	0
**1g**	38	59	0
**1g**	39	54	0
**1g**	40	48	0
**1g**	41	11	0
**1g**	42	68	0
**1g**	43	46	0
**1g**	44	21	13
**1g**	45	0	0
**1g**	46	0	0
**1g**	47	18	0
**1g**	48	8	24
**1g**	49	41	0
**1g**	50	51	0
**1g**	51	79	0
**1g**	52	58	0
**1g**	53	56	0
**1g**	54	0	0
**1g**	55	0	0
**1g**	56	0	0
**1g**	57	73	0
**1g**	58	55	0
**1g**	59	0	0
**1g**	60	0	0
**1g**	61	54	0
**1g**	62	0	0
**1g**	63	74	0
**1g**	64	0	0
**1g**	65	13	0
**1g**	66	55	0
**1g**	67	58	37
**1g**	68	66	0
**1g**	69	64	0
**1g**	70	62	0
**1g**	71	59	0
**1g**	72	3	0
**1g**	73	58	0
**1g**	74	62	0
**1g**	75	53	0
**1g**	76	56	0
**1g**	77	52	0
**1g**	78	40	34
**1g**	79	48	0
**1g**	80	59	0
**1g**	81	47	0
**1g**	82	57	0
**1g**	83	37	1
**1g**	84	54	0
**1g**	85	8	0
**1g**	86	57	0
**1g**	87	35	0
**1h**	1	58	8
**1h**	2	52	0
**1h**	3	64	60
**1h**	4	59	7
**1h**	5	2	0
**1h**	6	59	6
**1h**	7	60	0
**1h**	8	20	0
**1h**	9	55	6
**1h**	10	55	44

**1h**	11	55	0
**1h**	12	63	0
**1h**	13	59	0
**1h**	14	61	0
**1h**	15	0	0
**1h**	16	58	0
**1h**	17	60	6
**1h**	18	59	0
**1h**	19	54	7
**1h**	20	54	6
**1h**	21	59	5
**1h**	22	55	6
**1h**	23	72	42
**1h**	24	56	5
**1h**	25	51	5
**1h**	26	37	0
**1h**	27	67	0
**1h**	28	48	0
**1h**	29	58	38
**1h**	30	56	44
**1h**	31	6	0
**1h**	32	59	6
**1h**	33	17	0
**1h**	34	21	0
**1h**	35	50	0
**1h**	36	57	7
**1h**	37	52	37
**1h**	38	61	7
**1h**	39	55	32
**1h**	40	64	45
**1h**	41	58	42
**1h**	42	68	0
**1h**	43	58	42

**1h**	44	54	0
**1h**	45	6	25
**1h**	46	6	0
**1h**	47	19	0
**1h**	48	8	0
**1h**	49	47	7
**1h**	50	58	9
**1h**	51	63	7
**1h**	52	59	5
**1h**	53	61	6
**1h**	54	1	0
**1h**	55	22	0
**1h**	56	0	0
**1h**	57	54	6
**1h**	58	65	0
**1h**	59	3	0
**1h**	60	4	0
**1h**	61	55	0
**1h**	62	6	0
**1h**	63	55	0
**1h**	64	22	0
**1h**	65	6	0
**1h**	66	52	8
**1h**	67	57	8
**1h**	68	69	7
**1h**	69	60	8
**1h**	70	60	6
**1h**	71	61	6
**1h**	72	33	1
**1h**	73	60	6
**1h**	74	52	6
**1h**	75	60	8
**1h**	76	59	5

**1h**	77	55	43
**1h**	78	36	6
**1h**	79	53	6
**1h**	80	61	7
**1h**	81	54	7
**1h**	82	58	3
**1h**	83	28	0
**1h**	84	65	5
**1h**	85	8	2
**1h**	86	57	2
**1h**	87	33	4

Table S3. Data to support Figure 2B. Product peak area values were calculated by PyParse, an automated LCMS analysis software.

Figure 3

Product peak area by LCMS / %

	HCl salt			TFA salt	
**Library monomer**	**Standard**	**2 x EDC**	**2.5 eq. EDC**	**Standard**	**2.5 eq. EDC**
1	60	80	49	9	50
2	6	68	52	8	45
3	18	67	51	15	48
4	16	67	51	15	43
5	14	73	46	13	41
6	32	79	47	36	44
7	19	61	51	17	45
8	5	74	30	8	21
9	15	59	59	13	58
10	17	60	66	20	42
11	16	67	55	40	54
12	16	74	47	26	39
13	16	65	62	17	44
14	33	80	10	31	25
15	4	61	56	7	56
16	16	84	54	57	21
17	36	78	44	41	40
18	15	59	11	23	10
19	18	50	60	14	58
20	15	60	30	25	26
21	17	63	49	18	42
22	19	61	53	17	47
23	17	60	59	14	60
24	18	57	10	25	12
25	19	62	44	17	42
26	13	18	27	27	0
27	15	68	46	9	0
28	7	71	32	9	0
29	20	59	0	21	0
30	17	58	9	18	9
31	6	67	31	5	47
32	65	95	0	72	0
33	12	44		6	
34	3	50		5	
35	8	46		12	
36	14	59		15	
37	13	51		18	
38	10	51		16	
39	11	50		14	
40	17	44		27	
41	14	59		19	
42	4	53		14	
43	13	48		18	
44	19	49		64	
45	0	41		27	
46	6	56		11	
47	4	47		8	

48	5	39		11	
49	12	61		16	
50	16	3		46	
51	10	93		14	
52	16	65		18	
53	16	63		15	
54	9	45		16	
55	7	67		8	
56	3	46		8	
57	16	66		17	
58	14	65		22	
59	14	59		56	
60	9	81		13	
61	12	64		14	
62	32	83		15	
63	14	61		13	
64	11	75		12	
65	9	74			
66	13	55			
67	16	63			
68	18	72			
69	19	68			
70	14	63			
71	18	67			
72	16	68			
73	14	57			
74	17	65			
75	14	62			
76	15	66			
77	15	60			
78	3	62			
79	14	60			
80	17	61			
81	15	64			
82	17	17			
83	17	60			
84	16	0			
85	10	52			
86	18	0			
87	16	0			

Table S4. Data to support Figure 3. Product peak area values were calculated by PyParse, an automated LCMS analysis software.

Figure 4A

	Product peak area by LCMS / %			
	TFA salt		HCl salt	
Library monomer	Standard	3 minute premix	Standard	3 minute premix
1	9	15	12	12
2	8	42	17	45
3	15	44	25	47
4	15	13	13	11
5	13	51	25	52
6	36	58	48	64
7	17	44	28	49
8	8	28	11	29
9	13	40	25	48
10	20	46	34	50
11	40	15	32	14
12	26	45	47	54
13	17	48	31	53
14	31	10	21	8
15	7	30	11	32
16	57	61	23	68
17	41	65	54	68

18	23	44	35	48
19	14	44	29	56
20	25	43	39	52
21	18	54	36	57
22	17	41	32	49
23	14	44	29	51
24	25	44	37	48
25	17	43	28	46
26	27	21	25	15
27	9	26	17	32
28	9	18	13	23
29	21	45	32	55
30	18	46	30	51
31	5	20	9	24
32	72	71	75	80
33	6	11	30	14
34	5	18	9	18
35	12	38	23	44
36	15	11	13	10
37	18	43	28	50
38	16	40	28	48
39	14	43	26	50
40	27	49	37	55
41	19	0	26	58
42	14	25	20	29
43	18	45	31	65
44	64	60	67	55
45	27	31	31	19
46	11	24	15	24
47	8	21	14	26
48	11	2	1	3
49	16	45	29	48
50	46	26	33	23
51	14	46	32	54
52	18	42	34	50
53	15	45	27	53
54	16	37	21	44
55	8	23	12	24
56	8	24	13	27
57	17	46	30	46
58	22	47	37	50
59	56	26	50	21
60	13	28	17	30
61	14	52	28	53
62	15	61	21	65
63	13	42	21	50
64	12	57	13	62

Table S5. Data to support Figure 4A. Product peak area values were calculated by PyParse, an automated LCMS analysis software.

Figure 4B

	Product peak area by LCMS / %	
Library monomer	Standard	3 minute premix
1	14	15
2	34	33
3	36	37
4	33	33
5	34	33
6	26	23
7	36	35
8	24	23
9	55	53
10	29	49
11	40	40
12	35	34

13	35	35
14	24	23
15	39	36
16	17	17
17	35	32
18	13	13
19	48	45
20	23	24
21	34	31
22	36	37
23	19	19
24	62	58
25	32	32
26	36	33
27	34	31
28	35	34
29	16	16
30	20	21
31	34	34
32	73	76
33	23	8
34	28	28
35	31	30
36	31	32
37	36	36
38	33	36
39	29	18
40	17	17
41	33	34
42	41	39
43	33	33
44	17	17
45	35	32
46	35	34
47	36	40
48	29	32
49	13	13
50	12	13
51	30	30
52	37	38
53	20	20
54	60	48
55	18	19
56	1	2
57	37	34
58	33	30
59	28	31
60	18	20
61	35	34
62	23	24
63	55	53
64	48	50

Table S6. Data to support Figure 4B. Product peak area values were calculated by PyParse, an automated LCMS analysis software.

Figure 5A

Mixing condition	Salt	Monomer	DP / % 3	SM / % 1	Conversion
Mix dispense	TFA salt	1	60	0	100.0
		1	60	0	100.0
		2	70	0	100.0
		2	72	0	100.0
		3	61	0	100.0
		3	61	0	100.0
		4	77	0	100.0
		4	76	0	100.0

		5	59	0	100.0
		5	59	0	100.0
	HCl salt	1	36	0	100.0
		1	36	0	100.0
		2	46	0	100.0
		2	50	0	100.0
		3	38	0	100.0
		3	37	0	100.0
		4	65	0	100.0
		4	66	0	100.0
		5	66	0	100.0
		5	55	0	100.0
Centrifuge	TFA salt	1	19	29	39.6
		2	0	71	0.0
		3	0	26	0.0
		4	28	28	50.0
		5	0	57	0.0
Thermomixer - 24 h	TFA salt	1	0	50	0.0
		2	0	0	/
		3	0	48	0.0
		4	21	30	41.2
		5	0	25	0.0
Thermomixer - 72 h	TFA salt	1	34	0	100.0
		2	0	/	/
		3	0	34	0.0
		4	18	21	46.2
		5	21	28	42.9
Control	TFA salt	1	0	/	0.0
		2	0	/	0.0
		3	0	/	0.0
		4	0	/	0.0
		5	36	15	70.6

Table S7. Data to support Figure 5A. Conversion = product/product + amine; DP = desired product (PROTAC), SM = starting material (amine).

Figure 6A and 6B

		Product peak area by LCMS / %		
**Solvent**	**Monomer**	**Free amine**	**HCl salt**	**TFA salt**
**DMSO**	1	100	81.8	38.6
	2	100	63.2	31
	3	100	73.7	39
	4	100	72.7	45.8
	5	100	76.2	29.3
**DMF**	1	50	76.2	21.1
	2	100	100	54.2
	3	32.6	28.6	19.5
	4	40.1	25.6	32
	5	15.8	18.4	33.3
**DMA**	1	86.5	100	74.1
	2	100	97.6	80.6
	3	66.1	73.4	72.9
	4	59.5	61.1	80.6
	5	15.1	25	42.9
**NMP**	1	97.9	62.5	100
	2	100	100	100
	3	78.3	66.4	98.6
	4	88.3	70.5	98.8
	5	39.1	64.6	98.6

Table S8. Data to support Figure 6A and 6B.

Table 1

			1536-well plate			Batch scale			
PROTAC	Solvent	Salt	DP / % 3	SM / % 1	Conversion	DP / % 3	SM / % 1	Conversion	Yield / %
**3d**	DMSO	TFA	17	27	38.6	40	0	100	41
**3d**	DMSO	HCl	19	18	51.4	35	0	100	50
**3d**	DMSO	Free base	50	13	79.4	48	0	100	76
**3d**	DMF	TFA	3.5 [a]	13 [a]	21.1 [a]	43	0	100	40
**3d**	DMA	TFA	21.5 [a]	7.5 [a]	74.1 [a]	41	0	100	39
**3d**	NMP	TFA	36 [a]	0 [a]	100 [a]	34	0	100	31
**3b**	DMSO	HCl	12	4	75.0	48	0	100	47
**3c**	DMSO	Free base	47	0	100.0	33	0	100	42
**3a**	DMF	TFA	7.5 [a]	16 [a]	32.0 [a]	57	0	100	56
**3e**	NMP	HCl	25.5 [a]	14 [a]	64.6 [a]	31	0	100	41

Table S9. Table 1 containing additional data for calculation of conversion values. Amount of PROTAC product ***3a-e*** *and starting material* ***1b*** *were quantified by % peak area by LCMS; conversion = product/product + amine; batch scale performed on 46.4 μmol of amine* ***2a-e*** *(310-fold increase from 1536-well plate); yields were obtained after HPLC purification; DP = desired product (PROTAC), SM = starting material (amine); [a] mean calculated from N=2.*

References

1. Stevens, R.; Bendito-Moll, E.; Battersby, D. J.; Miah, A. H.; Wellaway, N.; Law, R. P.; Stacey, P.; Klimaszewska, D.; Macina, J. M.; Burley, G. A.; Harling, J. D., Integrated Direct-to-Biology Platform for the Nanoscale Synthesis and Biological Evaluation of PROTACs. *J. Med. Chem.* **2023,** *66* (22), 15437-52.
2. Zografou-Barredo, N. A.; Hallatt, A. J.; Goujon-Ricci, J.; Cano, C., A beginner's guide to current synthetic linker strategies towards VHL-recruiting PROTACs. *Bioorg. Med. Chem.* **2023,** *88-89*, 117334.
3. Sosic, I.; Bricelj, A.; Steinebach, C., E3 ligase ligand chemistries: from building blocks to protein degraders. *Chem. Soc. Rev.* **2022,** *51* (9), 3487-3534.
